# Effectiveness of psychological interventions in endometriosis: a systematic review with meta-analysis

**DOI:** 10.3389/fpsyg.2024.1457842

**Published:** 2024-10-28

**Authors:** Tasmania del Pino-Sedeño, María Cabrera-Maroto, Alejandra Abrante-Luis, Yadira González-Hernández, M Caridad Ortíz Herrera

**Affiliations:** ^1^Canary Islands Health Research Institute Foundation (FIISC), Tenerife, Spain; ^2^Evaluation Unit (SESCS), Canary Islands Health Service (SCS), Tenerife, Spain; ^3^Network for Research on Chronicity, Primary Care, and Health Promotion (RICAPPS), Tenerife, Spain; ^4^Faculty of Health Sciences, Universidad Europea de Canarias, Tenerife, Spain; ^5^Agencia Sanitaria Costa del Sol (ASCS), Marbella, Spain

**Keywords:** endometriosis, chronic pain, psychological interventions, quality of life, anxiety, depression

## Abstract

**Introduction:**

Endometriosis is a chronic gynecological disease associated with chronic debilitating pain, poor mental health and quality of life. The objective of this paper is to evaluate the effectiveness of psychological interventions aimed at improving the pain, quality of life and mental health of women with endometriosis.

**Methods:**

A systematic review (SR) of the literature with meta-analysis (MA) was carried out. MEDLINE, Embase, PsycINFO and CENTRAL were searched to locate Randomized Controlled Trials (RCTs). The risk of bias assessment of each study was conducted using the Cochrane Collaboration’s RoB 2.0 tool.

**Results:**

Seven RCTs were included (*N* = 757). The data obtained suggest that psychological interventions reduce dyspareunia [standardized mean difference (SMD): -0.54, 95% CI: −0.86, −0.22] and dyschezia [mean difference (MD): -2.90, 95% CI: −4.55, −1.26] and increase mental health levels (SMD: 0.70, 95% CI: 0. 42, 0.99); they also point to a large reduction in levels of trait anxiety (MD: -6.63, 95% CI: −8.27, −4.99) and depression (MD: -2.49, 95% CI: −3.20, −1.79), and a likely reduction in state anxiety (MD: -9.72, 95% CI: −13.11, −6.33) experienced by women with endometriosis. It was also identified that psychological interventions probably slightly reduce pelvic pain and may increase physical health. However, most of the included studies have a high overall risk of bias or have certain concerns, which limit conclusions about the certainty of the evidence.

**Discussion:**

The available evidence indicates that psychological interventions are effective in improving the pain, quality of life and mental health variables of women with endometriosis.

**Systematic review registration:**

https://www.crd.york.ac.uk/prospero/, CRD42024516100.

## 1 Introduction

Endometriosis is a gynecological disease in which endometrium-like tissue grows outside its normal anatomical location, causing a chronic inflammatory reaction (Leyland et al., 2010) that is associated with chronic debilitating pain and poor mental health (Evans et al., 2019). The causes of endometriosis are not fully understood. Although there are many theories about its origin, none of them can fully explain all aspects of the disease (Lamceva et al., 2023). It is estimated that endometriosis affects around 10% of women and girls of reproductive age worldwide, and it is observed in all social classes and ethnic groups (World Health Organization, 2023).

One of the biggest problems for women with the disease is the delay in diagnosis (Ruszała et al., 2022), which can take around 7 years to be identified (Zondervan et al., 2020). This may be due to the variability of symptoms, their non-specific nature and the difficulty in reaching a definitive diagnosis which, until recently, could only be made by surgical removal of tissue and pathological analysis (Kiesel and Sourouni, 2019; World Health Organization, 2023). However, nowadays, it is common practice to perform an ultrasound or MRI for diagnosis; reserving surgery for those patients with negative imaging results or in whom empirical treatment is unsuccessful (Lamceva et al., 2023; World Health Organization, 2023). However, while the diagnosis is being confirmed, women may experience persistent symptoms that affect their quality of life (QoL) and the disease may progress (Davenport et al., 2023).

Endometriosis can be classified into levels or grades based on the lesions caused, their location and their severity. The most used classification today is the one recommended by the American Society for Reproductive Medicine, which identifies four stages (I: minimal; II: mild; III: moderate; and IV: severe) (Practice Committee of the American Society for Reproductive Medicine, 2012). However, the stage of endometriosis does not correlate with the presence or severity of symptoms (National Guideline Alliance, 2017; Practice Committee of the American Society for Reproductive Medicine, 2012).

Endometriosis-related symptoms can affect women’s physical, psychological, and QoL areas (Van Niekerk et al., 2019). The physical symptoms of endometriosis vary depending on the person, and may include pain during menstruation (dysmenorrhea), pain during intercourse (dyspareunia), difficulty defecating (dyschezia), discomfort when urinating (dysuria), gastrointestinal problems, fatigue, pain headache, pelvic pain, lower abdominal pain, back pain, infertility, as well as a multiplicity of symptoms that are not specific (Gruber and Mechsner, 2021; Machairiotis et al., 2021; Prescott et al., 2016; Zondervan et al., 2020). However, endometriosis can also occur asymptomatically (Nnoaham et al., 2019).

Chronic pelvic pain is the main symptom of the disease, present in 80% of patients (Bulletti et al., 2010). The level of physical disability associated with endometriosis is primarily related to the impact of persistent pain that limits work, social, and daily living activities (Culley et al., 2013; Nnoaham et al., 2011).

As regards psychological symptoms, patients with endometriosis have a higher risk of developing depression, anxiety and stress (Donatti et al., 2022), among other conditions related to mental health (Delanerolle et al., 2021). Women with endometriosis have prevalence rates of 86% for depression, 29% for moderate to severe anxiety and 68% for mood disorders in general, which are significantly higher than the prevalence of these disorders in the general population (Chaman-Ara et al., 2017). Anxiety and depression symptoms are related to experienced chronic pain (Van Barneveld et al., 2022). Other psychological consequences of endometriosis include: feelings of worthlessness, helplessness, guilt, isolation, impaired interpersonal relationships, sleep problems, and self-directed violence (Ruszała et al., 2022). Additionally, another problem that contributes to worsening the emotional state of some patients is infertility which can be caused by the disease (Ruszała et al., 2022).

Women with endometriosis have a significant decrease in QoL compared to women without endometriosis (Bourdel et al., 2019). These patients are affected in their abilities to perform daily activities, exercise motherhood, maintain satisfactory sexual relationships, maintain employment and productivity, study or interact with friends, among others (Aredo et al., 2017; Hansen et al., 2017; Ruszała et al., 2022).

Since no curative treatment is available, care must be directed toward symptom management (World Health Organization, 2023). Typical interventions include laparoscopic surgery to excise the lesions and hormonal, anti-inflammatory, and analgesic medication (Becker et al., 2022). However, many women derive only limited or intermittent benefits from treatment (Becker et al., 2017). Numerous studies have shown the high possibility of increased pain and relapse when discontinuing these medications, in addition to the fact that current medical treatments can cause unwanted side effects, including weight gain, hirsutism, acne, vaginal atrophy, breast atrophy, hot flushes, decreased libido, fatigue, nausea and vomiting (Samami et al., 2023). Also noteworthy are the drugs from the gonadotropin-releasing hormone (GnRH) analog group that cause a suppression of ovarian activity with significant menopausal symptoms in many patients, which further negatively affects their QoL (Samami et al., 2023). This is why a large number of women seek other health approaches and non-pharmacological techniques to address their disease (Evans et al., 2019; Schwartz et al., 2019).

In this regard, evidence-based multidisciplinary care is necessary to address endometriosis (Ruszała et al., 2022). This interdisciplinary management of the disease should reinforce support for mental health in patient care, beyond pain management (Brasil et al., 2020).

The role of psychological interventions in the treatment of symptoms and psychological distress related to endometriosis has been reported (Chaman-Ara et al., 2017; Chiantera et al., 2017), which is why their incorporation is proposed in the planning of the treatment offered to these women (Van Niekerk et al., 2019). Various psychological interventions, such as progressive muscle relaxation (PMR), mindfulness, psychotherapy, and cognitive behavioral therapy (CBT), among others, have shown potential in improving QoL and alleviating clinical symptoms (Donatti et al., 2022; Samami et al., 2023). However, their efficacy in endometriosis requires further exploration.

Cognitive Behavioral Therapy (CBT) is one of the most researched psychological interventions, combining cognitive and behavioral strategies to modify maladaptive cognitive misperceptions and maladaptive behaviors (Beck, 1995; Dobson and Dozois, 2019). Rooted in learning and cognitive theories (Bandura, 1969; Yates, 1970), CBT aims to modify unhelpful thoughts and behaviors, using techniques like exposure therapy to reduce avoidance and foster adaptive responses (Abramowitz, 2013; Carpenter et al., 2019). Systematic reviews highlighted both the strengths and limitations of CBT in managing chronic pain. While CBT has been effective in reducing insomnia and improving social participation and self-efficacy in patients with chronic low back pain and musculoskeletal conditions, its effects on pain intensity and fatigue are less pronounced (Liu et al., 2024; Salazar-Méndez et al., 2024; Selvanathan et al., 2021; Yang et al., 2022; Zhang et al., 2023). Additionally, CBT has proven effective in decreasing headache frequency and intensity in migraine sufferers, though further research is needed (Bae et al., 2021).

Jacobson’s PMR technique is a systematic method used to achieve a deep state of relaxation. It has proven effective in reducing stress, anxiety, and depression in adults; as well as in improving cancer-related fatigue, anxiety, depression and sleep quality in patients with cancer (Wang et al., 2024a), including those experiencing chronic pain (Muhammad Khir et al., 2024; Steen et al., 2024; Tan et al., 2022).

Mindfulness is another approach that trains individuals to remain in the present moment and engage with their experiences nonjudgmentally. Mindfulness practices include attention training, body scanning, and sitting meditation, which help patients build awareness and acceptance of their experiences (Kabat-Zinn, 1990). Mindfulness-based interventions (MBIs) have a demonstrated efficacy in improving psychological well-being across diverse clinical populations. For example, MBIs have been shown to have short-term benefits in reducing anxiety and depression and improving quality of life in patients with inflammatory bowel disease (Qian and Zhang, 2024). In breast cancer patients, MBI has led to significant improvements in coping abilities, emotional regulation, and a reduction in adverse emotional states (Wang et al., 2024b; Wu et al., 2022). Furthermore, MBIs have been shown to reduce pain intensity in individuals with chronic pain conditions, such as chronic low back pain, and have been recommended as part of a multidisciplinary approach, including pelvic floor physical therapy, for managing chronic pelvic pain in women (Bittelbrunn et al., 2023; Paschali et al., 2024).

Acceptance and Commitment Therapy (ACT) is a therapy designed to enhance psychological flexibility by helping individuals connect with and accept their present psychological or emotional experiences while living in alignment with their values (Hayes et al., 2012). This therapy targets six core processes—acceptance, cognitive defusion, being present, self-as-context, values, and committed action—that are relevant across various clinical conditions (Hayes et al., 2012). In the context of chronic pain, ACT has been shown to significantly reduce cognitive fusion, a key factor in the persistence of pain, thereby improving overall psychological well-being and QoL (Sanduvete-Chaves et al., 2024). Meta-analyses further support ACT’s effectiveness in alleviating pain-related distress and enhancing functional outcomes across different chronic pain conditions, with particularly significant short-term benefits observed in patients with chronic headaches and fibromyalgia (Lai et al., 2023; Ye et al., 2024).

While these psychological interventions show promise, their specific effectiveness in managing endometriosis-related symptoms remains uncertain, necessitating further research to assess their impact on pain, QoL, and mental health in this population.

The objective of the present systematic review (SR) with meta-analysis (MA) is to identify, evaluate and synthesize the available scientific evidence on the effectiveness of psychological interventions aimed at improving the pain, quality of life and mental health variables of women diagnosed with endometriosis. The hypotheses of the present SR are that psychological interventions will help to: (1) alleviate the sensation of pain, (2) enhance the quality of life, and (3) improve the mental health of women affected by this condition.

## 2 Methods

An SR with MA was conducted according to the methodology set out in the Cochrane Handbook (Higgins et al., 2023). This review reports following the guidelines of the PRISMA statement (Page et al., 2021). The protocol of the present review has been registered in Prospero (CRD42024516100).

### 2.1 Eligibility criteria

Studies were selected that evaluated the effectiveness of psychological interventions in women diagnosed with endometriosis and that met the selection criteria below.

Only randomized controlled trials (RCTs) were included.

By patient type, women diagnosed with endometriosis were included without age limit.

By intervention, those studies that implemented psychological programs or interventions were included and any type of comparator was considered (no treatment, waiting list or alternative treatments).

Regarding the outcome measures, physical and psychological effects such as pain, QoL, and symptoms of anxiety or depression were included, which were evaluated through standardized scales.

As regards language, studies published in both Spanish and English (languages mastered by the authors) were considered.

### 2.2 Information sources

A search was conducted in the MEDLINE, Embase, PsycINFO and CENTRAL databases (October 10, 2023). The search was completed with manual examination of the bibliographic list of the SRs found in the search.

### 2.3 Search strategy

A search strategy without a date limit was developed around the terms: Endometriosis, Behavior Therapy, Cognitive Behavioral Therapy, Psychological Techniques, Psychology, Psychotherapy, Acceptance and Commitment Therapy, Behavioral Disciplines and Activities, Mental Health Services and Dialectical Behavior Therapy. This search strategy was designed in MEDLINE and was subsequently adapted to the other consulted databases. The complete strategy can be consulted in Supplementary Table S1.

### 2.4 Study selection process

The bibliographic references obtained in the different databases were imported into RefWork, where duplicates were automatically eliminated. The unique references were then exported to a Microsoft Excel 2016 sheet (Microsoft Corporation) for selection. In the first phase, references were selected by title and abstract. In the second phase, the preselected references were selected in full text taking into account the selection criteria described above. Both phases were performed by two reviewers independently. All discrepancies were resolved through discussion.

### 2.5 Data extraction process

Data extraction from the included studies was performed using a data extraction sheet in Excel 2016 format (Microsoft Corporation). Data extraction from the rest of the studies was carried out by two reviewers independently.

### 2.6 Data list

The extracted data included the identification and design of the study (title, authors, year of publication, conflict of interest, funding, country, context, objective, design, number of centers, number of groups and follow-up periods), the characteristics of the participants (clinical condition, inclusion and exclusion criteria, number of participants and losses, and sociodemographic and clinical characteristics), the interventions (description, method, provider, number of sessions, duration and periodicity) and the outcome measures (instruments, evaluation points and conclusion). The statistical results presented in each study were extracted in detail [means (M), standard deviations (SD), *p*-values and sample sizes (N)].

### 2.7 Assessment of the risk of Bias of individual studies

The risk of bias assessment of each study was performed with the RoB 2.0 tool developed by the Cochrane Collaboration for RCTs (Higgins et al., 2019). A pilot test was conducted with one of the studies by both reviewers and, subsequently, the rest of the studies were evaluated. The entire process was carried out independently.

### 2.8 Effect measures

The outcome measures analyzed were continuous. Therefore, the extent of the effects of the psychological interventions evaluated in terms of pain, QoL, anxiety and depression were estimated as mean difference (MD) or standardized mean difference (SMD), with its 95% confidence interval (95% CI).

In addition, SMDs were computed to standardize results, allowing for comparison across studies regardless of the measurement units used and quantifying the intervention’s impact as a standardized measure of effect size. Guidelines for interpreting SMDs are as follows: values of <0.40 are considered small, 0.40 to 0.70 moderate, and > 0.70 large (Higgins et al., 2023; Schünemann et al., 2024).

### 2.9 Synthesis methods

The information collected was synthesized narratively with tabulation of the results of each included study. When pooling was not possible, the effects were described narratively. Furthermore, a quantitative synthesis using MA was performed when the reported data were combinable and the studies were clinically and methodologically homogeneous. The MD or SMD of the outcome measures evaluated were estimated using the inverse variance method (Egger et al., 2008; Fleiss, 1993). Heterogeneity in the MA results was evaluated graphically by presenting the estimated effects and their 95%CI of each study in a forest plot, as well as by Higgins’ *I*^2^ statistic (Higgins, 2003). Following the recommendations, the presence of substantial heterogeneity was considered when the I^2^ value was greater than 50% (Deeks et al., 2023). In this case, instead of a fixed effects model, a random effects model was applied. In the presence of high and unexplained heterogeneity (*I*^2^ ≥ 90%), MA was not performed and evidence synthesis was reported narratively. Likewise, the individual contribution of each study to the observed heterogeneity was analyzed by means of a sensitivity analysis, excluding one study at a time in cases in which a clinical or methodological basis was found. For all of the aforementioned, the statistical program Review Manager Version 5.4 was used (RevMan 5) (The Cochrane Collaboration, 2020).

The following potential confounders were considered: baseline level of pain, type of endometriosis, type of intervention, number of sessions and risk of bias of individual studies.

Subgroup analyses were performed by group when it was possible. Meta-regression analyses were limited because of the small number of studies evaluated.

### 2.10 Publication Bias assessment

Assessment of the risk of publication bias was performed by visual inspection of the funnel plots of each analysis and further explored by computing the Egger test (Egger et al., 1997), with a significance level set at 0.05. Funnel plots were performed using RevMan, and the Egger tests were conducted using the metabias command in STATA version 17.

### 2.11 Certainty of evidence

Certainty of the evidence was judged for all outcomes using the Grading of Recommendations Assessment, Development and Evaluation working group methodology (GRADE Working Group), across the domains of risk of bias, inconsistency, indirectness, imprecision, publication bias, large effect, dose response and all plausible residual confounding (Balshem et al., 2011). Certainty was adjudicated as high, moderate, low or very low (Atkins et al., 2004). A Summary of Findings (SoF) table was prepared to present the main comparisons and outcomes (Guyatt et al., 2013).

## 3 Results

**Figure 1:**
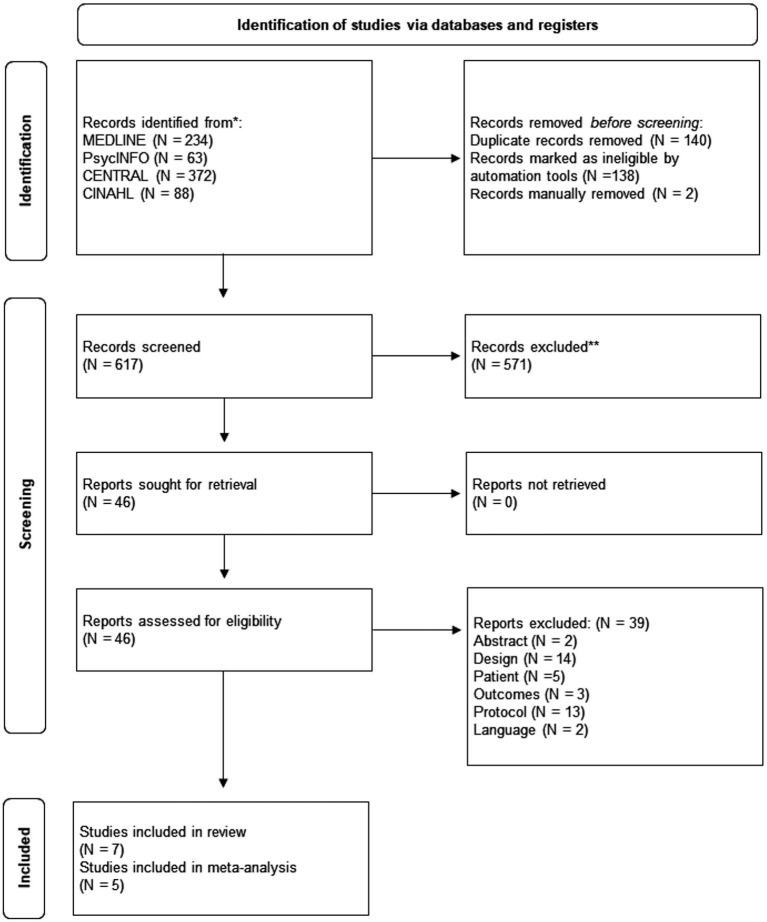
Flow diagram of the selection process.

A total of 757 references were identified by the search in the different databases, of which 46 articles were selected for full-text evaluation once duplicates were eliminated and the title and abstract selection was carried out. Finally, after complete evaluation, seven studies were included (Farshi et al., 2020; Hansen et al., 2023; Meissner et al., 2016; Moreira et al., 2022; Tajik et al., 2022; Zandi et al., 2023; Zhao et al., 2012). Manual searches did not provide any additional references (see Figure 1). The reasons for exclusion can be found in Supplementary Table S2.

### 3.1 Characteristics of the included studies

The seven included studies were published in English between 2012 and 2023 and conducted in China (Zhao et al., 2012), Germany (Meissner et al., 2016), Denmark (Hansen et al., 2023), Brazil (Moreira et al., 2022) and Iran (Farshi et al., 2020; Tajik et al., 2022; Zandi et al., 2023). All were RCTs with simple randomization and two intervention arms, except for Hansen et al. which had three arms (Hansen et al., 2023). The follow-up periods were variable. The minimum follow-up was carried out post-intervention (Hansen et al., 2023; Zhao et al., 2012) and the maximum after 2 years (Meissner et al., 2016), with the majority being studies that performed at least one evaluation per month, after completing the intervention (Farshi et al., 2020; Moreira et al., 2022; Tajik et al., 2022; Zandi et al., 2023). Information on the general characteristics of the studies can be found in Table 1.

**Table 1:** Characteristics of included studies.

First author, year	Context	Follow-up time (post intervention)	CI	Funding
Farshi et al. (2020)	Teaching and treatment center	4 weeks	No	Tabriz University of Medical Sciences
Hansen et al. (2023)	Specialized outpatient clinics for endometriosis	0, 2 weeks	No	TrygFonden, Ladywalk, and the Danish Endometriosis Patient Association
Meissner et al. (2016)	Institute of Medical Psychology, Departments of Neuroradiology, Obstetrics and Gynecology, Neurology clinic, Department of Gynecologic Endocrinology and Fertility Disorder	3, 6, 24 months	No	Horst Görtz Foundation, Theophrastus Foundation and Schweizer-Arau Foundation
Moreira et al. (2022)	Endometriosis Outpatient Clinic	1, 4 weeks	No	No
Tajik et al. (2022)	Fertility clinic	1, 2 months	No	Medical University of Tarbiat Modares
Zandi et al. (2023)	Fertility clinic	4, 8 weeks	No	No
Zhao et al. (2012)	Obstetrics and Gynecology Departments	0 weeks	NR	NR

CI, conflict of interest; NA, Not applicable; NR, Not reported.

Regarding the participants, 520 women were recruited, with an average of 74, a minimum of 58 (Hansen et al., 2023) and a maximum of 100 patients per study (Zhao et al., 2012), and 65 losses (12.50%). The clinical condition of the participants was in all cases a diagnosis of endometriosis, although in some studies the presence of pain was specified (Hansen et al., 2023; Meissner et al., 2016), the medical treatment received was specified (Tajik et al., 2022; Zandi et al., 2023; Zhao et al., 2012) or the level of affectation was specified (Moreira et al., 2022; Tajik et al., 2022). The mean age was 34.7 years (SD = 1.56). The selected studies included diagnoses by MRI laparoscopy, which favors the diagnostic accuracy of the included patients. The demographic and clinical characteristics of the participants in each study are shown in Table 2.

**Table 2:** Selection criteria and baseline characteristics of participants.

First author, year	Clinical Condition	Inclusion criteria	Exclusion	N		Previous treatment/medication *N* (%)	Actual treatment/medication *N* (%)	Marital status *N* (%)	Biological children	Age Mean (SD)	N			
				R	P						I	L	C	L
Farshi et al. (2020)	Endometriosis	1) Residing in Tabriz 2) at least secondary school education degrees 3) Diagnosed with endometriosis via laparoscopy during the past 5 years 4) 15–45 years 5) accessible via landline phone or cellphone numbers	1) Any condition that increased the risk of anxiety and depression 2) Antidepressants (3 months) 3) Malignancies 4) Severe depression and very severe anxiety 5) Recent trauma 6) Speech or hearing disorders 7) Being pregnant 8) A history of past mental illness or hospitalization for this reason	76	3	1) Laparoscopy: 61 (80.26%) 2) Laparoscopy+ medical: 11 (14.47%) 3) Laparoscopy+ medical+ Herbal: 3 (3.95%) 4) Laparoscopy+ herbal: 1 (1.32%)	NR	1) Widow: 1 (1.32%) 2) Divorced: 79 (21%) 3) Married: 60 (78.95%) 4) Single: 8; (10.53%)	1) 0: 20 (26.32%) 2) 1: 23 (30.26%) 3) 2 or more: 22 (28.95%)	34.4^†^ (NR)	38	0	38	3
Hansen et al. (2023)	Endometriosis and chronic pelvic pain	1) 18–47 years 2) Surgery or MRI-confirmed endometriosis diagnosis 3) Moderate to severe chronic pelvic pain 4) relevant clinical and surgical treatment according to the ESHRE guidelines 5) Willingness to spend 30–45 min on housework 5–7 days a week for 10 weeks	1) Other serious physical pain diseases 2) Severe psychiatric diagnosis 3) pregnancy or planned 4) Estimated lack of mental or physical surplus to start a psychological treatment or linguistic or cultural barriers	58	16	1) Removal of endometriosis lesions: 48 (88.88%^†^) 2) No treatment: 52 (96.29%^†^) 3) Pain medication: 52 (96.3%^†^) 4) Physical treatment: 25 (46.3%^†^) 5) Psychological treatment: 4 (7.41%^†^) 6) Psychological treatment: 14 (25.93%^†^)	1) No treatment: 3 (5.56%^†^) 2) No treatment: 45 (83.33%^†^) 3) Pain medication: 43 (79.63%^†^)	1) Married/living together 33 (61.11%^†^) 2) Single: 15 (27.78%^†^) 3) Others: 6 (1.11%^†^)	1) 0: 35 (64.81%^†^) 2)1: 9 (16.67%^†^) 3) 2: 7 (12.97%^†^) 4) 3: 3 (5.56%^†^)	31.82^†^ (NR)	20	6	19	6
Meissner et al. (2016)	Endometriosis and chronic pelvic pain	1) 18–40 years 2) A history of histologically verified endometriosis 3) Chronic pelvic pain	1) Hormonal treatment 2) Drug or alcohol addiction 3) Pregnancy 4) Insufficient knowledge of German 5) Contraindications for MRI	67	11	Surgical treatment during last laparoscopy: Complete removal of endometriosis lesions: 35 (52,24%) Incomplete or no removal of endometriosis lesions: 32 (47,76%)	Use of analgesics NSAIDs: 41 (61.19%) Opioids: 5 (7.46%) Other: 17 (25.37%)	NR	NR	35.6 (NR)	35	5	32	6
Moreira et al. (2022)	Deep endometriosis	1) 18–50 years 2) ≥1 deep endometriotic nodules evaluated by MRI 3) Endometriosis-related pain of moderate to severe intensity (≥6 months)	1) Current or past 6-month meditation-related practices 2) Other treatment initiation or treatment change (3 months before and during the trial) 3) Psychotic symptoms 4) Current suicidal ideation 5) Malignant lesions 6) Pregnancy 7) Inability to understand assessment or treatment instructions	63	17	NR	Dienogest: 48 (76.19%) COC: 15 (23.81%)	1) Divorced: 4 (6.35%) 2) Married: 29 (46.03%) 3) Single: 29 (46.03%)	NR	36.15^†^ (NR)	31	9	32	12
Tajik et al. (2022)	Peritoneal or superficial endometriosis + medical treatment (COC + GnRH)	1) Married 2) 18–45 years 3) Having sexual intercourse in the last 8 weeks	1) A known underlying disease other than endometriosis 2) A history of mental illness 3) Partner addicted to drugs or alcohol 4) A stressful accident in the past month 5) Taking drugs that affect sexual function 6) Urinary tract infection, vaginitis, cervicitis, active sores or genital lesions 7) A history of being sexually assaulted	80	0	NR	NR	1) Married: 80 (100%)	NR	35.61 (4.42)	40	0	40	0
Zandi et al. (2023)	Endometriosis	1) 15–45 years 2) Married and living with husband 3) Confirmed diagnosis of endometriosis by laparoscopy 4) Volunteering to participate in the study 5) Not having a history of psychological problems or chronic diseases 6) Ability to use the internet	1) Being absent for two or more sessions	76	5	1) yes: 65 (85.53%^†^) 2) no: 11 (14.47%^†^)	NR	1) Married: 76 (100%)	1) 0: 49 (64.47%^†^) 2) 1: 21 (27.63%^†^) 3) ≥2: 6 (7.90%^†^)	34.6^†^ (NR)	38	3	38	2
Zhao et al. (2012)	Endometriosis + GnRH	1) 18–48 years 2) Endometriosis verified by laparoscopy or laparotomy and confirmed by histology 3) Complaining of dysmenorrhea, dyspareunia and/or pelvic pain 4) Having failed COC therapy 5) An above-elementary school education 6) Able to communicate clearly and give informed consent	1) Previously surgically treated for endometriosis 2) Previously treated with a GnRH 3) A family or personal history of mental illness 4) Severe cognitive impairment 5) Concurrent oncologic or psychiatric diseases 6) Treatment for anxiety or depression	100	13	COC	NR	1) Married: 53 (60.9%) 2) Single: 34 (49.1%)	NR	NR	50	8	50	5

C, Control; COC, combined oral contraceptives; ESHRE, European Society of Human Reproduction and Embryology; GnRH, gonadotropin- releasing hormone analogs; I, Intervention; L, Loss; N, number of patients; NA, Not applicable; NR, Not reported; NSAIDs, nonsteroidal anti-inflammatory drugs; MRI, magnetic resonance imaging; R, recruited; Tx, treatment. ^†^Own calculation.

The psychological interventions evaluated in the included studies were PMR training (Zhao et al., 2012); psychotherapy with somatosensory stimulation (Meissner et al., 2016) and training in sensory focus techniques and sexual position change (Tajik et al., 2022); the MYENDO Program, based on mindfulness and acceptance-based psychological intervention (Hansen et al., 2023) and a brief intervention based on mindfulness (Moreira et al., 2022); a non-specific psychological intervention for endometriosis that included patient education, group therapy, relaxation, and guided physical training (Hansen et al., 2023); education based on the theory of planned behavior (Zandi et al., 2023) and a self-care counselling group (Farshi et al., 2020). In most studies, the intervention was conducted by the researchers (Farshi et al., 2020; Tajik et al., 2022; Zandi et al., 2023; Zhao et al., 2012), in one study the provider was a psychologist (Hansen et al., 2023) and in two it was conducted by professionals specialized in the technique used (Meissner et al., 2016; Moreira et al., 2022). For the most part, the intervention was weekly, with an average duration of eight weeks. The minimum number of sessions was one (Tajik et al., 2022) and the maximum was 24 (Zhao et al., 2012). The control group received the usual medical treatment or remained on the waiting list.

Regarding the outcome measures, four studies evaluated pain (Hansen et al., 2023; Meissner et al., 2016; Moreira et al., 2022; Tajik et al., 2022), six QoL (Farshi et al., 2020; Hansen et al., 2023; Meissner et al., 2016; Moreira et al., 2022; Zandi et al., 2023; Zhao et al., 2012) and three evaluated anxiety and depression (Farshi et al., 2020; Meissner et al., 2016; Zhao et al., 2012). Detailed information on the intervention and main outcome measures by study can be found in Table 3.

**Table 3:** Characteristics of the psychological interventions and control groups in the included studies.

First author, year	Intervention	Intervention deliverer	Sessions			Total length	Control group	Pain	QoL	Anxiety	Depression
			*N*	Duration (minutes)	Periodicity						
Farshi et al. (2020)	Self-care group counselling	Researcher	7	60–90	1/week	7 weeks	Routine care	NA	SF-36	STAI	BDI
Hansen et al. (2023)	MYENDO. Specific mindfulness- and acceptance-based psychological intervention	Psychologist	10	180	1/week	10 weeks	Waiting list, medical treatment as usual	NRS	EHP-30	NA	NA
	Non-specific psychological intervention (relaxation and guided physical training)	Psychologist	10	NR	1/week	10 weeks					
Meissner et al. (2016)	Psychotherapy with somatosensory stimulation	A medical specialist for psychosomatic medicine and traditional Chinese medicine	NR	30–60	NR	NR	Waiting list control, cared for by the study gynecologists	NRS	SF-12	HADS, STAI	HADS
Moreira et al. (2022)	Brief Mindfulness-Based Intervention + conventional medical treatment	An experienced mindfulness instructor	4	90	1/week	4 weeks +3 home exercise instructions	Standard medical care (hormonal therapy and analgesics)	NRS	SF-36	NA	NA
Tajik et al. (2022)	Sensate focus technique and sexual positions	Researcher	1	120	NA	1 session	Routine care	VAS	NA	NA	NA
Zandi et al. (2023)	Education based on the theory of planned behavior	Researcher	4	90–120	1/week	4 weeks	Routine hospital care	ERHQ	NA	NA	NA
Zhao et al. (2012)	Progressive muscle relaxation training + GnRH (1 dose of depot leuprolide, 11.25 mg)	Researcher	24	40	2/week	12 weeks	GnRH (1 dose of depot leuprolide, 11.25 mg)	NA	SF-36	STAI	HADS

BDI, Beck Depression Inventory; EHP-30 QoL, Danish version of The Endometriosis Health Profile 30 questionnaire; GnRH, gonadotropin- releasing hormone analogs; HADS, Hospital Anxiety and Depression Scale; NA, Not applicable; NR, Not reported; NRS, 0–10-point numeric rating scale; SF-36, Short Form-36 Health Survey; SF-12, Short Form-12 Health Survey; STAI, State–trait Anxiety Inventory; VAS, Visual analog scale.

### 3.2 Risk of Bias of individual studies

**Figure 2:**
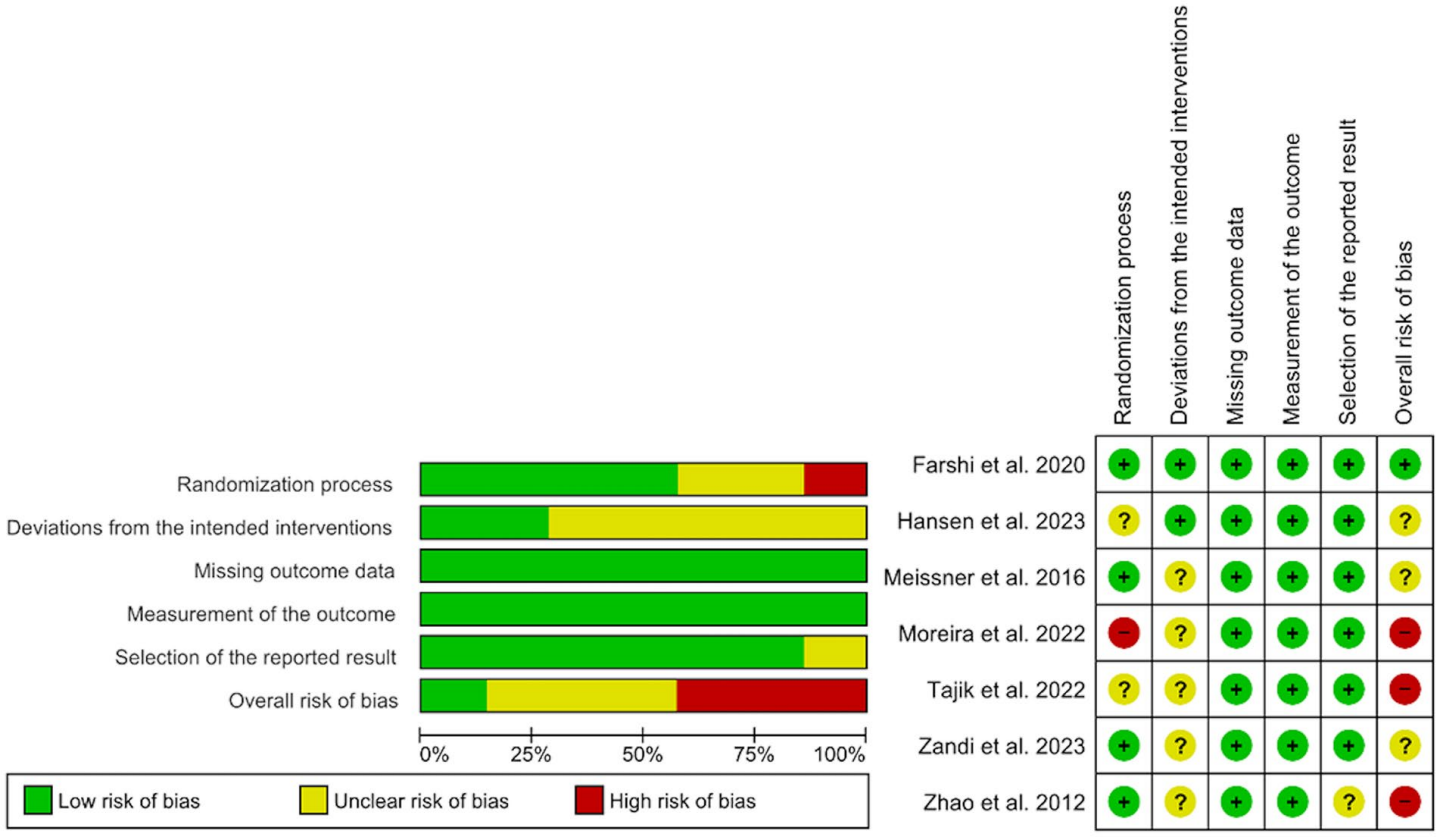
Risk of bias in included studies.

Three of the studies present a high risk of bias (Moreira et al., 2022; Tajik et al., 2022; Zhao et al., 2012), three of them suggest certain concerns, one regarding the risk of selection bias (Hansen et al., 2023) and the other two regarding the risk of performance bias (Meissner et al., 2016; Moreira et al., 2022), and only one study presents a low risk of bias in all domains (Farshi et al., 2020). Detailed judgments for each of the risk of bias domains are shown in Figure 2.

More specifically, three studies raise concerns about the risk of bias in the randomization process. Hansen et al. (2023) did not specify whether the allocation sequence was random, and Tajik et al. (2022) did not report if the allocation sequence was adequately concealed, resulting in an unclear risk for both studies. Furthermore, Moreira et al. (2022) did not clarify whether the allocation sequence was randomized, and baseline differences between intervention groups suggest a high risk of bias in this domain.

In relation to deviations from the intended interventions, in all studies, participants or carers were aware of the intervention received. Besides which, in some studies, an intention-to-treat analysis was not applied. Consequently, most studies were judged to raise concerns in this domain.

Finally, Zhao et al. (2012) presents an unclear risk of selective reporting, as no protocol was available to confirm that the results were aligned with a pre-specified analysis.

### 3.3 Certainty of evidence

The overall quality of evidence was very low. The evidence profile for psychological interventions vs. control outcomes showed that the quality of evidence was moderate to very low (Supplementary Table S3).

### 3.4 Results of individual studies and results of the synthesis

The results of the individual studies can be consulted in Supplementary Tables S4–S7. Of the total number of studies included, only five could be included in the MA (Farshi et al., 2020; Meissner et al., 2016; Moreira et al., 2022; Tajik et al., 2022; Zhao et al., 2012). The results are shown below. The results of all meta-analyses and sensitive analyses performed are available in Supplementary Table S8.

#### 3.4.1 Pain (certainty of the evidence: moderate ⊕ ⊕ ⊕ ⊖)

Of the four studies that evaluated changes in pain levels (Hansen et al., 2023; Meissner et al., 2016; Moreira et al., 2022; Tajik et al., 2022), three studied dyspareunia (Meissner et al., 2016; Moreira et al., 2022; Tajik et al., 2022), two dyschezia (Meissner et al., 2016; Moreira et al., 2022) and two pelvic pain (Meissner et al., 2016; Moreira et al., 2022), which were meta-analyzed.

**Figure 3:**
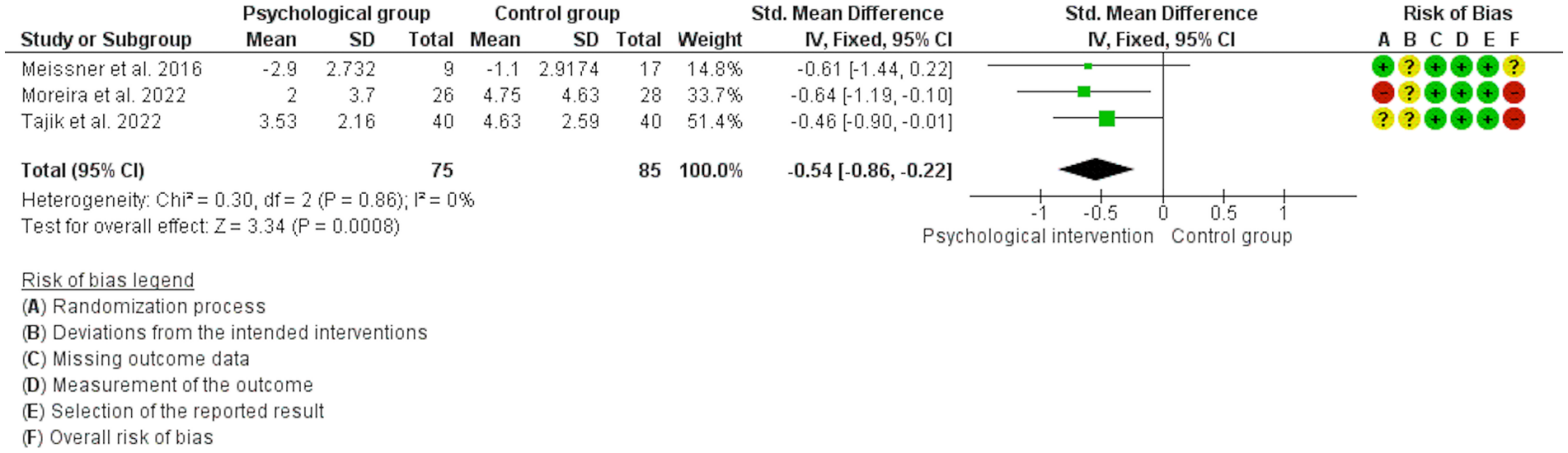
Forest plot for the effect of psychological interventions on dyspareunia.

**Figure 4:**
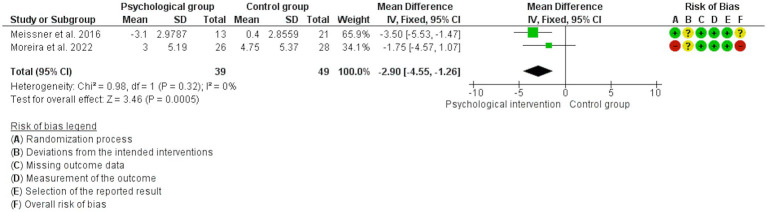
Forest plot for the effect of psychological interventions on dyschezia.

**Figure 5:**
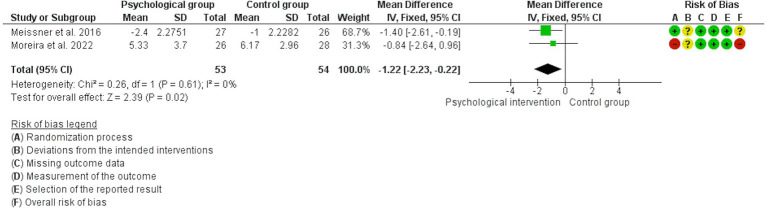
Forest plot for the effect of psychological interventions on pelvic pain.

The analyses showed that psychological interventions reduce the levels of dyspareunia (SMD: -0.54, 95% CI: −0.86, −0.22; *I*^2^ = 0%; *N* = 160; number of studies [*K*] = 3; see Figure 3) and the levels of dyschezia evaluated with the NRS scale (MD: -2.90, 95% CI: −4.55, −1.26; *I*^2^ = 0%; *N* = 88; *K* = 2; see Figure 4) versus usual treatment or the waiting list. In relation to the levels of pelvic pain, the psychological intervention was found to result in a slight reduction (MD: -1.22, 95% CI: −2.23, −0.22; *I*^2^ = 0%; *N* = 107; *K* = 2; see Figure 5).

#### 3.4.2 Quality of life (certainty of the evidence: very low ⊕ ⊕ ⊕ ⊖/moderate ⊕ ⊕ ⊕ ⊖)

Of the six studies that evaluated different domains related to QoL (Farshi et al., 2020; Hansen et al., 2023; Meissner et al., 2016; Moreira et al., 2022; Zandi et al., 2023; Zhao et al., 2012), four assessed mental health (Farshi et al., 2020; Meissner et al., 2016; Moreira et al., 2022; Zhao et al., 2012), two physical health (Farshi et al., 2020; Meissner et al., 2016), and two general health, vitality, social function, emotional role, physical role and physical functioning (Moreira et al., 2022; Zhao et al., 2012). All of them were initially meta-analyzed.

High heterogeneity was detected (*I*^2^ = 90%) in the analyses of the mental health component. In the sensitivity analysis it was observed that the study by Farshi et al. (2020), in which patients in the intervention group received advice on self-care, provided all of the heterogeneity. However, even eliminating this study, a moderate effect remains in favor of the guided psychological intervention that resulted in an increase in mental health compared to the waiting list or usual treatment (SMD: 0.70, 95% CI: 0.42, 0.99; *I*^2^ = 0%; *N* = 201; *K* = 3; see Supplementary Figure S1). Subgroup analysis confirmed that this heterogeneity was related to the type of treatment received (guided psychological intervention vs. self-care counselling; *p* < 0.01).

The analyses showed no statistically significant differences between the intervention and control groups in the remaining meta-analyzed QoL dimensions. Therefore, evidence suggests that psychological interventions may result in little or no difference in social functioning (MD: -4.47, 95% CI: −26.29, 17.35; *I*^2^ = 84%; *N* = 141; *K* = 2), the emotional role (MD: -15.98, 95% CI: −35.22, 3.27; *I*^2^ = 68%; *N* = 141; *K* = 2), or physical functioning (MD: 8.11, 95% CI: −5.61, 21.83; I^2^ = 57%; *N* = 141; *K* = 2) and probably produces little or no difference in the physical role (MD: 10.98, 95% CI: - 7.52, 29.49; *I*^2^ = 40%; *N* = 141; *K* = 2). The heterogeneity presented in these outcomes was not related to the intervention received or risk of bias. The forest plots can be consulted in Supplementary Figures S2–S5.

High heterogeneity was detected in the analyses of the physical health component (*I*^2^ = 98%), the vitality component (*I*^2^ = 92%), and the general health component evaluated by the SF-36 (*I*^2^ = 97%). This heterogeneity was not attributable to the type of intervention received or to risk of bias, leading to the decision not to present quantitative results for any of these components.

#### 3.4.3 Anxiety (certainty of the evidence: low ⊕ ⊕ ⊕ ⊖/moderate ⊕ ⊕ ⊕ ⊖)

Three studies evaluated changes in trait anxiety assessed with the STAI (Farshi et al., 2020; Meissner et al., 2016; Zhao et al., 2012), which were meta-analyzed.

The analyses showed that psychological interventions result in a large reduction (SMD: −1.04) of trait anxiety scores (MD: -6.63, 95% CI: −8.27, −4.99; *I*^2^ = 46%; *N* = 216; *K* = 3; see Supplementary Figure S6), compared to usual treatment or the waiting list.

**Figure 6:**
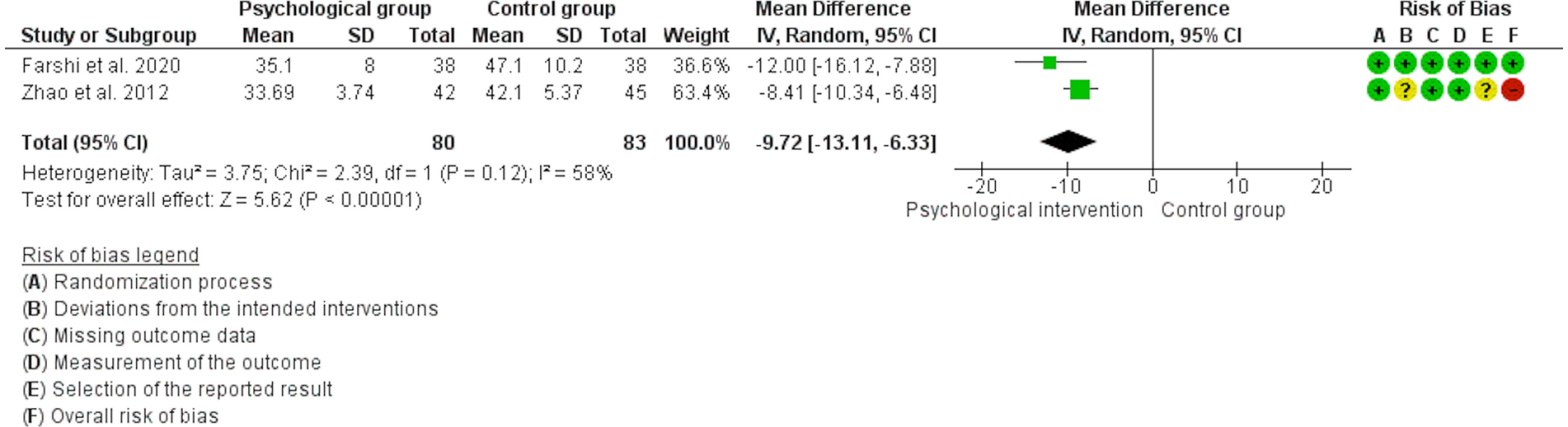
Forest plot for the effect of psychological interventions on state anxiety.

Two of the three previous studies reported data on changes in state anxiety assessed with the STAI (Farshi et al., 2020; Zhao et al., 2012). The analyses showed that the psychological intervention probably reduces state anxiety compared to the control group (MD: -9.72, 95% CI: −13.11, −6.33; *I*^2^ = 58%; *N* = 163; *K* = 2; see Figure 6). The heterogeneity was not associated with the type of intervention received or the risk of bias.

#### 3.4.4 Depression (certainty of the evidence: moderate ⊕ ⊕ ⊕ ⊖)

**Figure 7:**
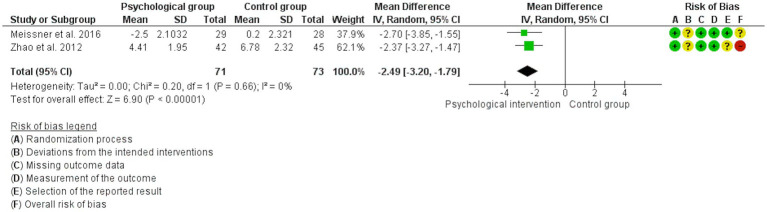
Forest plot for the effect of psychological interventions on depression.

Three studies analyzed changes in depression levels (Farshi et al., 2020; Meissner et al., 2016; Zhao et al., 2012). However, a high rate of heterogeneity was detected (*I*^2^ = 90%). The subgroup analysis showed that this was due to the type of treatment received (guided psychological intervention vs. self-care counselling) (*p* < 0.01). In the sensitivity analysis, the study by Farshi et al. (2020) was found to be the source of heterogeneity. After eliminating this study from the analysis, a large reduction (SMD: −1.14) in depression levels was observed in favor of guided psychological intervention (MD: -2.49, 95% CI: −3.20, −1.79; *I*^2^ = 0%; *N* = 144; *K* = 2; see Figure 7).

### 3.5 Publication Bias

Visual exploration of the funnel plots and the results of the Egger tests did not reveal any evidence of publication bias in the evaluated measures. These can be consulted in Supplementary Figures S7–S17 and Supplementary Table S8, respectively.

## 4 Discussion

The present SR evaluates the effectiveness of various psychological interventions on the mental health and pain management of women diagnosed with endometriosis. By including a range of approaches, such as mindfulness, acceptance-based psychological intervention, or PMR, the SR provides a comprehensive understanding of their impact. Additionally, by examining both mental health outcomes, like anxiety and depression, alongside physical outcomes, such as pain reduction, it offers an integrated perspective on how these interventions may contribute to the overall well-being of women with endometriosis. The findings underscore the potential role of psychological therapies as part of a broader treatment approach for managing both the mental and physical aspects of the condition.

The data obtained suggest that psychological interventions probably reduce pain levels (dyspareunia and dyschezia) and improve mental health. The data also point to a likely large reduction in levels of trait anxiety and depression, and a likely reduction in state anxiety experienced by women with endometriosis. It was also found that psychological interventions probably slightly reduce pelvic pain and may increase physical health. Other results indicate that psychological interventions probably make little or no difference to the physical role and may result in little or no difference in social function, emotional role, or physical functioning. The evidence on the effect of psychological interventions on general health and vitality in women diagnosed with endometriosis is currently uncertain.

Regarding the pain experienced by women with endometriosis, the analyses conclude that psychotherapy involving somatosensory stimulation, brief mindfulness-based interventions, and the techniques of sensory focus and change of sexual position have a positive effect on the levels of dyspareunia experienced by women with endometriosis. Similarly, levels of dyschezia and pelvic pain are lower after receiving psychotherapy with somatosensory stimulation and mindfulness-based interventions. Regarding overall pain, psychotherapy with somatosensory stimulation, the MYENDO program, based on mindfulness and acceptance, and non-specific psychological intervention including patient education, group therapy, relaxation and guided physical training seem to have positive effects. The results of the individual studies, such as Meissner et al. (2016), showed a statistically significant improvement in overall maximum pain and overall average pain in the psychological intervention group with somatosensory stimulation three months after the intervention, although its effect was less in the follow-ups at 6 and 24 months. In the study by Hansen et al. (2023), a reduction in the levels of pain intensity and unpleasantness was observed after the MYENDO intervention, compared to the non-specific psychological intervention and the group of patients on the waiting list. However, no statistically significant differences were found for other types of endometriosis-related pain outcomes, such as dysmenorrhea, dysuria (Moreira et al., 2022), or vaginal pressure pain threshold (Hansen et al., 2023). In general, these findings support that concluded in previous SRs in which MA was not carried out and other designs were considered, but suggested that cognitive behavioral therapy (Donatti et al., 2022; Samami et al., 2023; Van Niekerk et al., 2019), acceptance and commitment therapy (Van Niekerk et al., 2019), mindfulness-based interventions (Hilton et al., 2017; Samami et al., 2023; Van Niekerk et al., 2019), psychoeducation (Samami et al., 2023) and interventions that include physical components (Evans et al., 2019; Fernández-Pérez et al., 2023; Gonçalves et al., 2017), improve pain levels in endometriosis patients.

The above is an important implication since pain is one of the main symptoms of the disease, present in 80% of patients (Bulletti et al., 2010), which markedly affects their daily life (Della Corte et al., 2020; Dowding et al., 2024; Samami et al., 2023) and that is also related to other psychological variables such as depression and anxiety (Van Barneveld et al., 2022), so given its potential benefits and the absence of expected adverse effects compared to pharmacological and surgical treatments, the psychological interventions carried out should include or contemplate some of these treatment options.

Concerning QoL, the analyses in the present SR with MA indicate that psychological interventions may result in little or no difference in social functioning, emotional role, physical functioning, and probably produce minimal differences in physical role. Moreover, the evidence remains highly uncertain regarding the effects of psychological interventions on general health and vitality (Moreira et al., 2022; Zhao et al., 2012). However, the analyses also show that self-care counselling, psychotherapy with somatosensory stimulation, intervention based on mindfulness, PMR training, the MYENDO program, based on mindfulness and acceptance-based psychological intervention, and psychological intervention including patient education, group therapy, relaxation and guided physical training, and education based on the theory of planned behavior can have a positive effect on domains related to QoL such as mental health. In addition, the results of the individual studies suggest that psychological interventions can enhance physical health (Farshi et al., 2020; Meissner et al., 2016), as well as improve domains and areas such as control, emotional well-being, and social support (Hansen et al., 2023), reproductive health (Zandi et al., 2023), and overall QoL (Zhao et al., 2012). These results are in line with previous SR conclusions, in which other designs considered therapy (Donatti et al., 2022; Van Niekerk et al., 2019), but which also pointed out aspects such as acceptance of pain and coping strategies as important elements in the intervention (Bullo and Hearn, 2021; González-Echevarría et al., 2019). The fact that QoL can be improved by training pain management and emotional regulation strategies (Barberis et al., 2023; Márki et al., 2017, 2022) is reflected in the overall results of the present work, since in all the studies in which other mental health measures were evaluated, there was an improvement in QoL domains. This suggests that the design of interventions aimed at improving QoL in women with endometriosis should consider and even prioritize among its objectives the improvement of other variables such as emotional state or pain management.

With respect to anxiety, the present analyses show that self-care counselling, brief interventions based on mindfulness and PMR show benefits in both trait anxiety and state anxiety, as measured by the STAI. Besides which, the findings of Meissner et al. (2016), which evaluated anxiety levels using the Hospital Anxiety and Depression Scale, support these results. This supports the conclusions of previous SRs in which the role of psychoeducation (Van Niekerk et al., 2019) and other psychological techniques (Evans et al., 2019) were highlighted as treatments to improve anxiety in women with endometriosis. The heterogeneity introduced in the analysis of state anxiety by the study of Zhao et al. (2012), who used PMR, reported a greater effect, this could be due to the greater number of sessions in the treatment group compared to the rest of the studies, so this could be a factor to consider in the implementation of psychological intervention programs for these women.

As regards depression, both psychotherapy with somatosensory stimulation and PMR showed positive effects, unlike the self-care counselling group proposed by Farshi et al. (2020). This contrasts with the conclusions reached by Van Niekerk et al. (2019), in which they highlighted the positive effects of psychoeducation and Evans et al. (2019), in which they recommended support groups to reduce depression, but supports previous SRs in which the role of cognitive behavioral therapy was underlined (Donatti et al., 2022) as well as interventions including physical components (Evans et al., 2019). It would be desirable to have more evidence in SRs that include MA in order to come to more solid conclusions in this regard. In addition to being able to assess, if not dispensing with surgical or hormonal treatments with significant adverse effects, at least the reduction or delay of their requirements.

The data obtained show, therefore, that therapies combining physical and psychological aspects are those that deliver the best results (Donatti et al., 2022; Evans et al., 2019; Kirca and Celik, 2023), as well as influencing one of the variables of interest, which is pain. These results can also be observed in other diseases with common characteristics, such as fibromyalgia (Islam et al., 2022; Leça and Tavares, 2022; Theadom et al., 2015; Williams et al., 2020), cancer-related pain (Liu et al., 2022) or multiple sclerosis (Hadoush et al., 2022), where pain is, in turn, a fundamental part of the disease and treatment process.

The interrelationship between the pain experienced by women with endometriosis, QoL, and other mental health variables, along with the previously discussed findings, support the effectiveness of psychological interventions in addressing these variables.

### 4.1 Strengths and limitations

The present SR has a series of strengths, such as: (1) compared to other SRs, the present SR included only the best possible evidence for the evaluation of the effectiveness of intervention programs (RCTs) and incorporated MA in the synthesis of results, which provides robustness to its conclusions; (2) a rigorous and transparent methodology was used in accordance with the principles of science and the standards of SRs and MAs; and (3) the steps followed have been detailed, guaranteeing replicability.

Regarding the weaknesses, the following can be highlighted: (1) since it was carried out in a limited number of databases and without analysis of the possible gray literature, the bibliographic search could not identify other relevant studies, however, the manual searches performed in the SR suggest the possibility of having located all the available published evidence; (2) only studies published in English and/or Spanish were taken into account, so some studies were left out of this SR; (3) the scarcity of evidence, small sample sizes, and heterogeneity between the selected studies for some of the outcomes studied, which sometimes leads to inconsistent and imprecise results and limits the possibility of conducting meta-regression analyses and exploring the effect of potential confounders such as baseline pain level, type of endometriosis, or the number of intervention sessions.

## 5 Conclusion

In conclusion, the evidence currently available indicates that psychological interventions have moderate positive effects on pain levels (dyspareunia and dyschezia), and moderate to strong positive effects on the anxiety and depression experienced by women with endometriosis, as well as on different components of QoL such as mental health or physical health, plus a small positive effect on pelvic pain. Therefore, the treatment of these women needs to go beyond medical and surgical management and include validated psychological treatments. However, although some recommendations have been highlighted to guide interventions in this regard, a greater number of studies are needed to reach more solid conclusions.

## Data Availability

The original contributions presented in the study are included in the article/Supplementary material, further inquiries can be directed to the corresponding author/s.
