# Supplementary material for: Effectiveness of psychological interventions in endometriosis: a systematic review with meta-analysis
Source: Front Psychol. 2024 Oct 28;15:1457842. doi: 10.3389/fpsyg.2024.1457842 (PMC11551779; doi:10.3389/fpsyg.2024.1457842)
Supplement: Supplementary file 1 [file Data_Sheet_1.DOCX]

Supplementary Material

**Supplementary Figure 1** Forest Plot for the Effect of Psychological Interventions on Quality of Life: Mental Health (sensitive analysis)


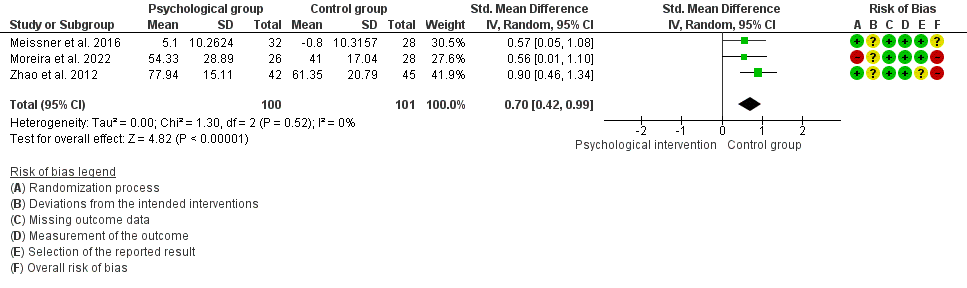


**Supplementary Figure 2** Forest Plot for the Effect of Psychological Interventions on Quality of Life: Social Function


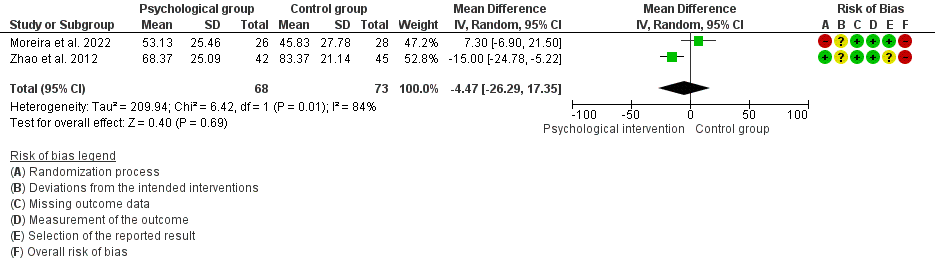


**Supplementary Figure 3** Forest Plot for the Effect of Psychological Interventions on Quality of Life: Emotional Role


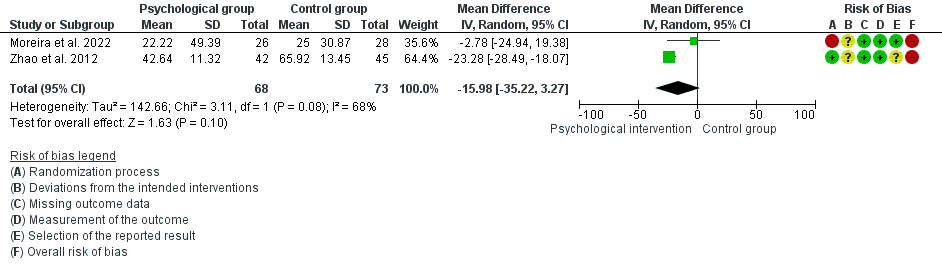


**Supplementary Figure 4** Forest Plot for the Effect of Psychological Interventions on Quality of Life: Physical Functioning


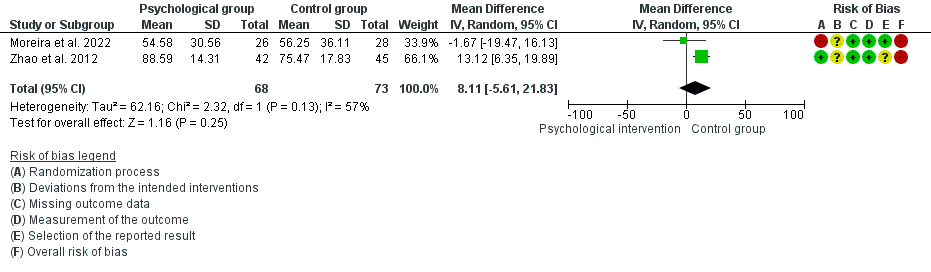


**Supplementary Figure 5** Forest Plot for the Effect of Psychological Interventions on Quality of Life: Physical Role


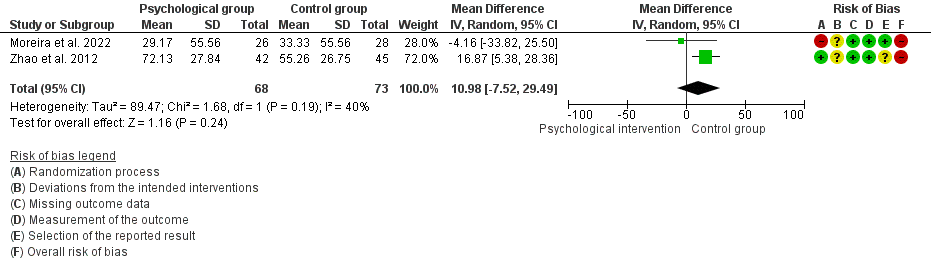


**Supplementary Figure 6** Forest Plot for the Effect of Psychological Interventions on Trait Anxiety


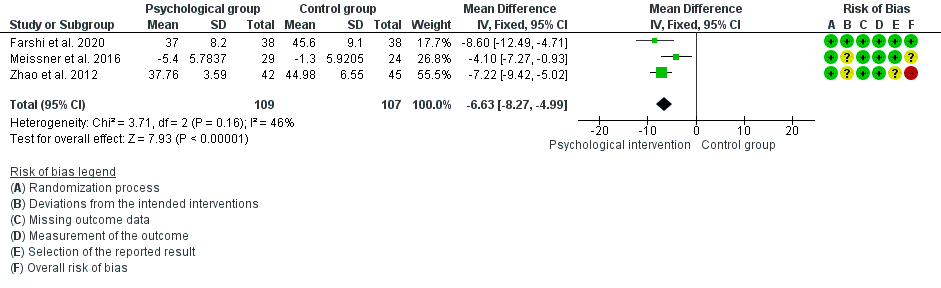


**Supplementary Figure 7** Funnel Plot for Pain: Dyspareunia

**
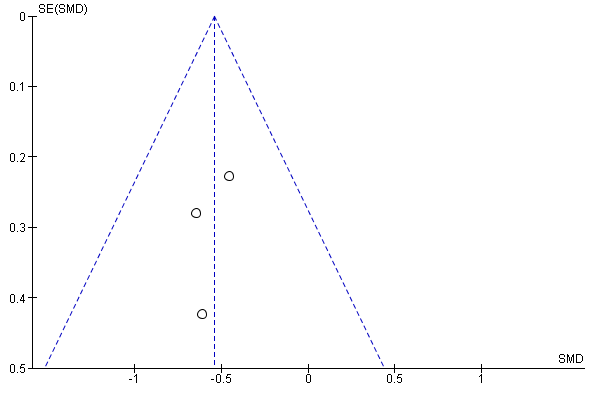
**

**Supplementary Figure 8** Funnel Plot for Pain: Dyschezia


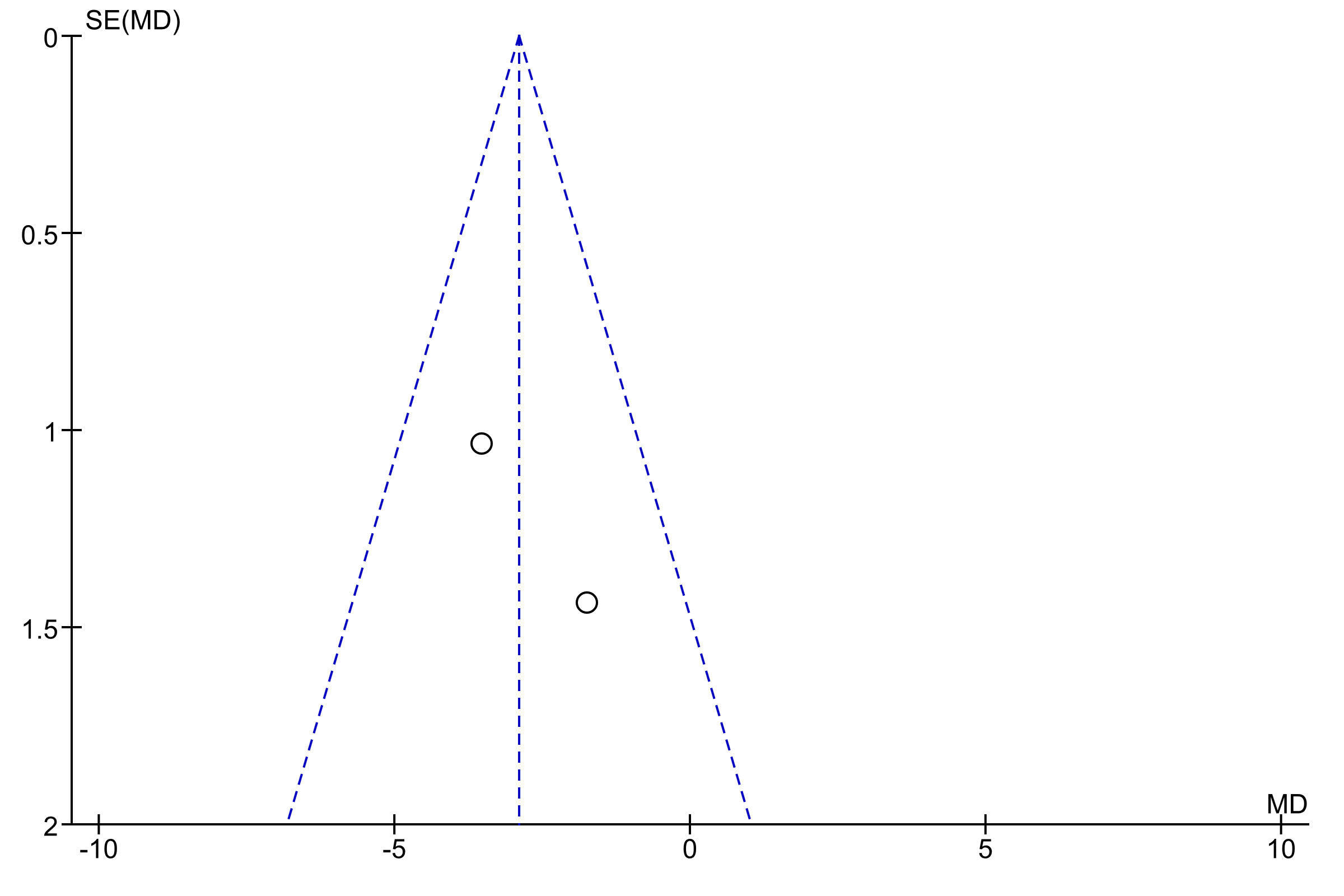


**Supplementary Figure 9** Funnel Plot for Pain: Pelvic Pain


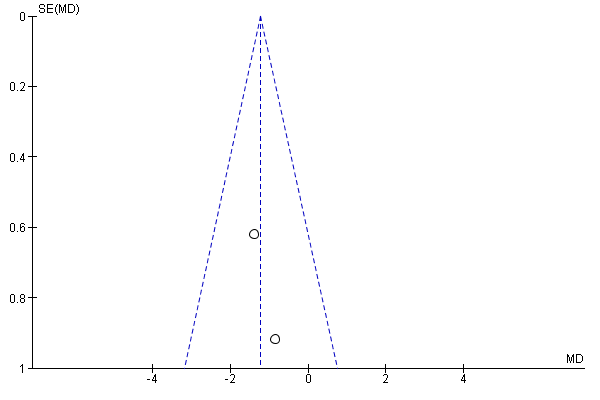


**Supplementary Figure 10** Funnel Plot for Quality of Life: Mental Health


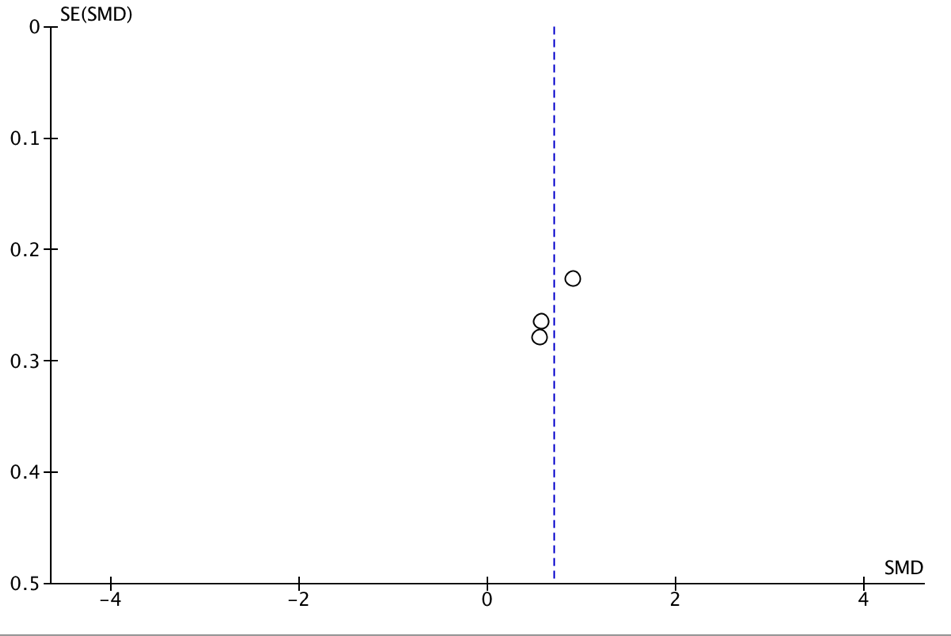


**Supplementary Figure 11** Funnel Plot for Quality of Life: Social Function


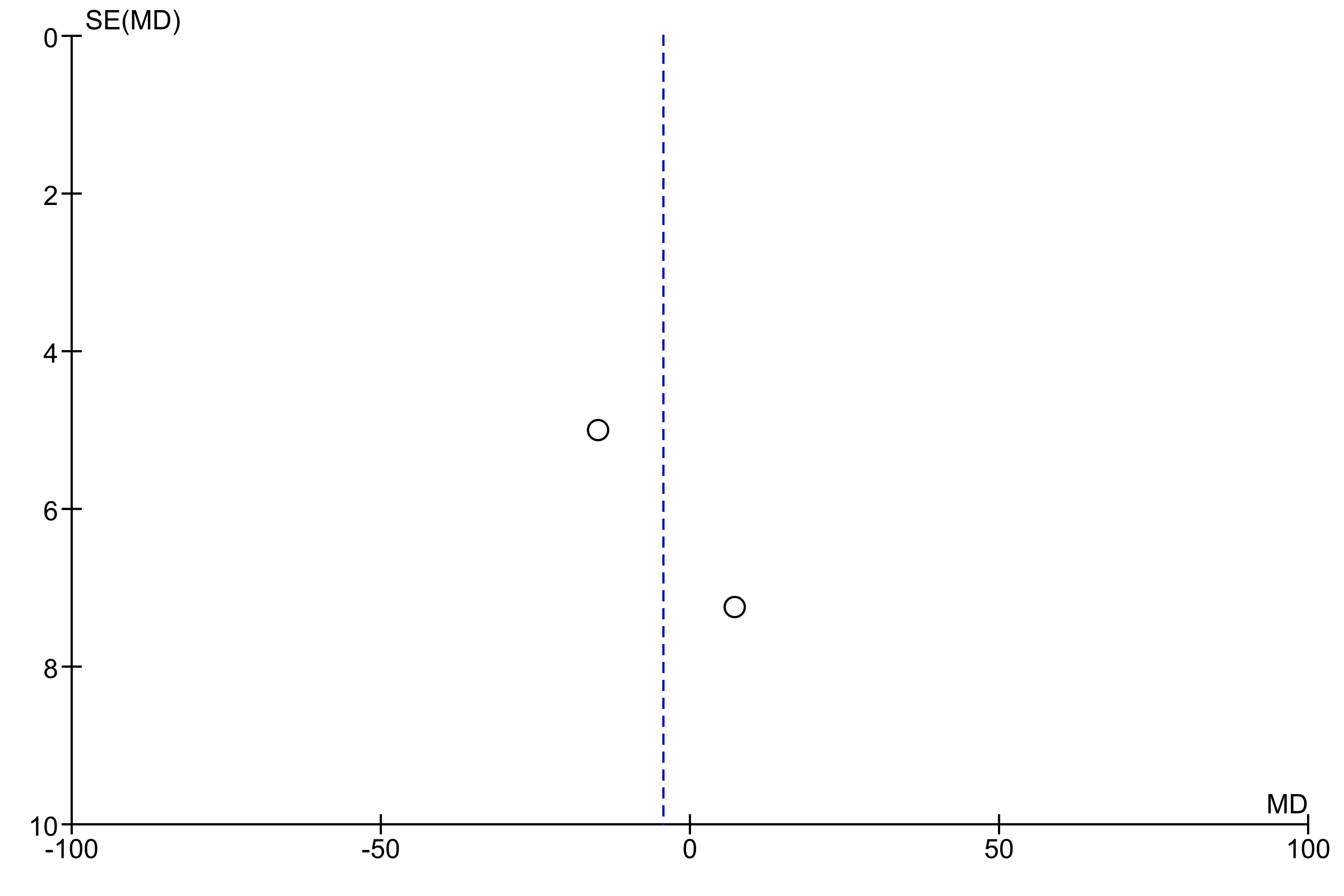


**Supplementary Figure 12** Funnel Plot for Quality of Life: Emotional Role


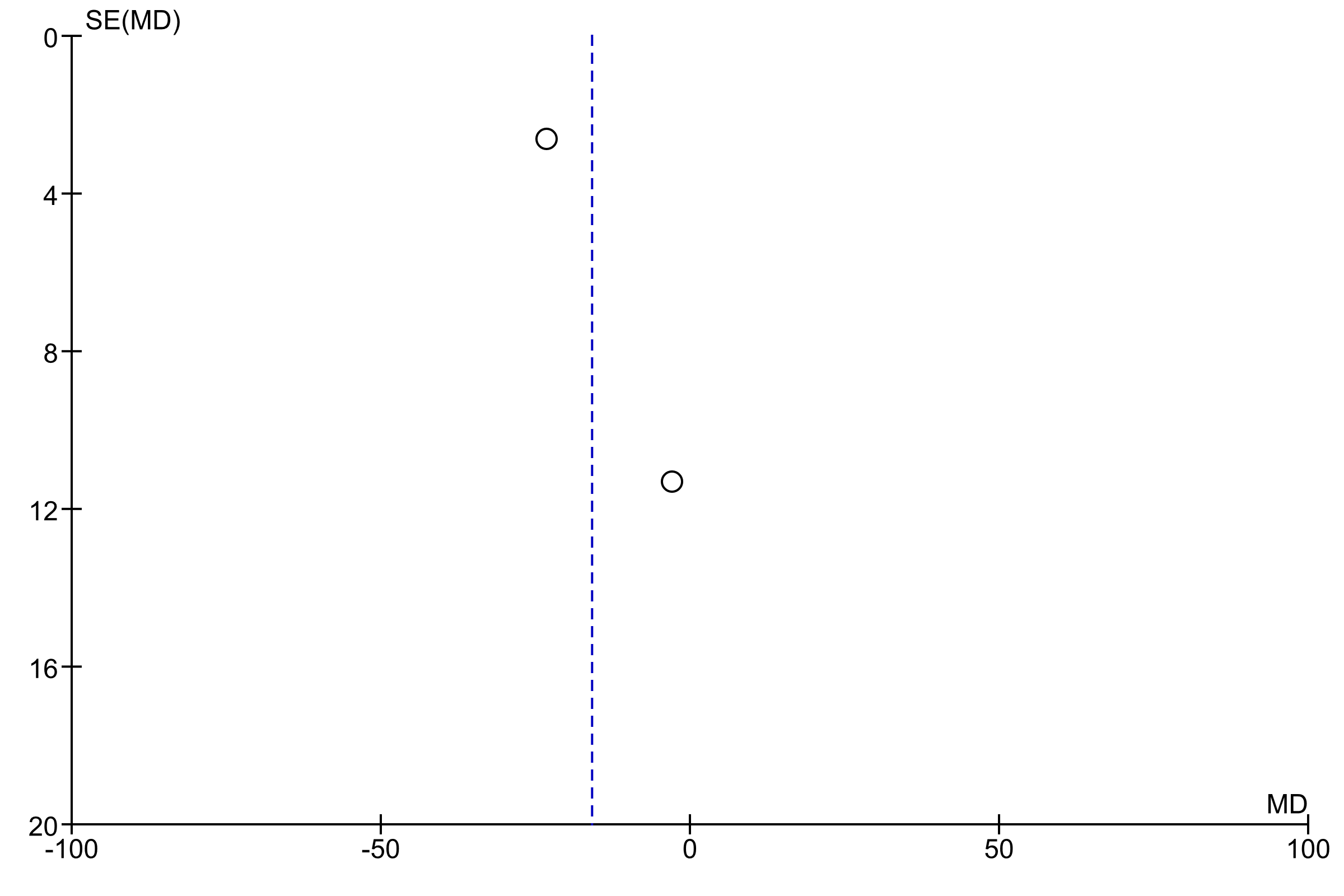


**Supplementary Figure 13** Funnel Plot for Quality of Life: Physical Functioning


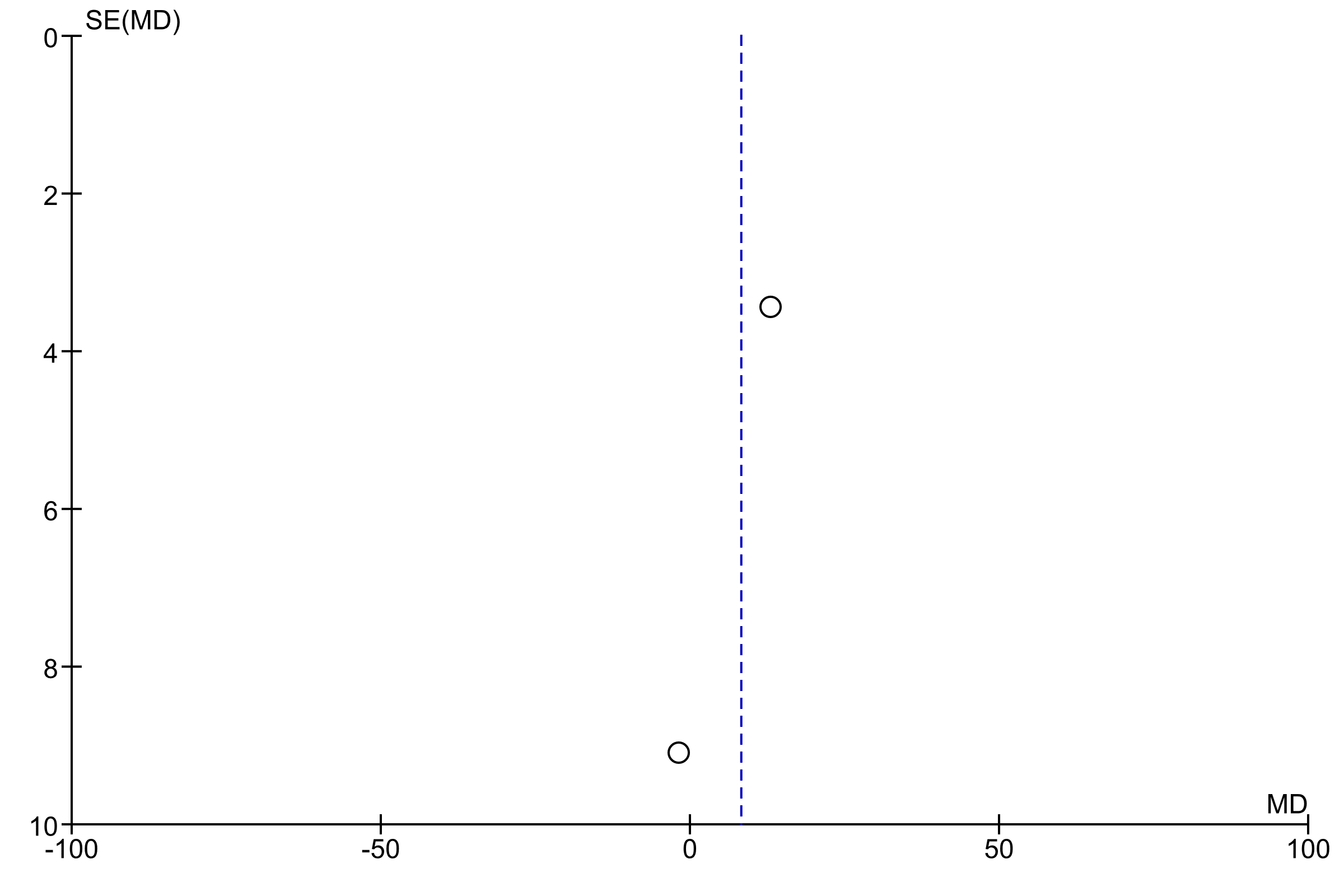


**Supplementary Figure 14** Funnel Plot for Quality of Life: Physical Role


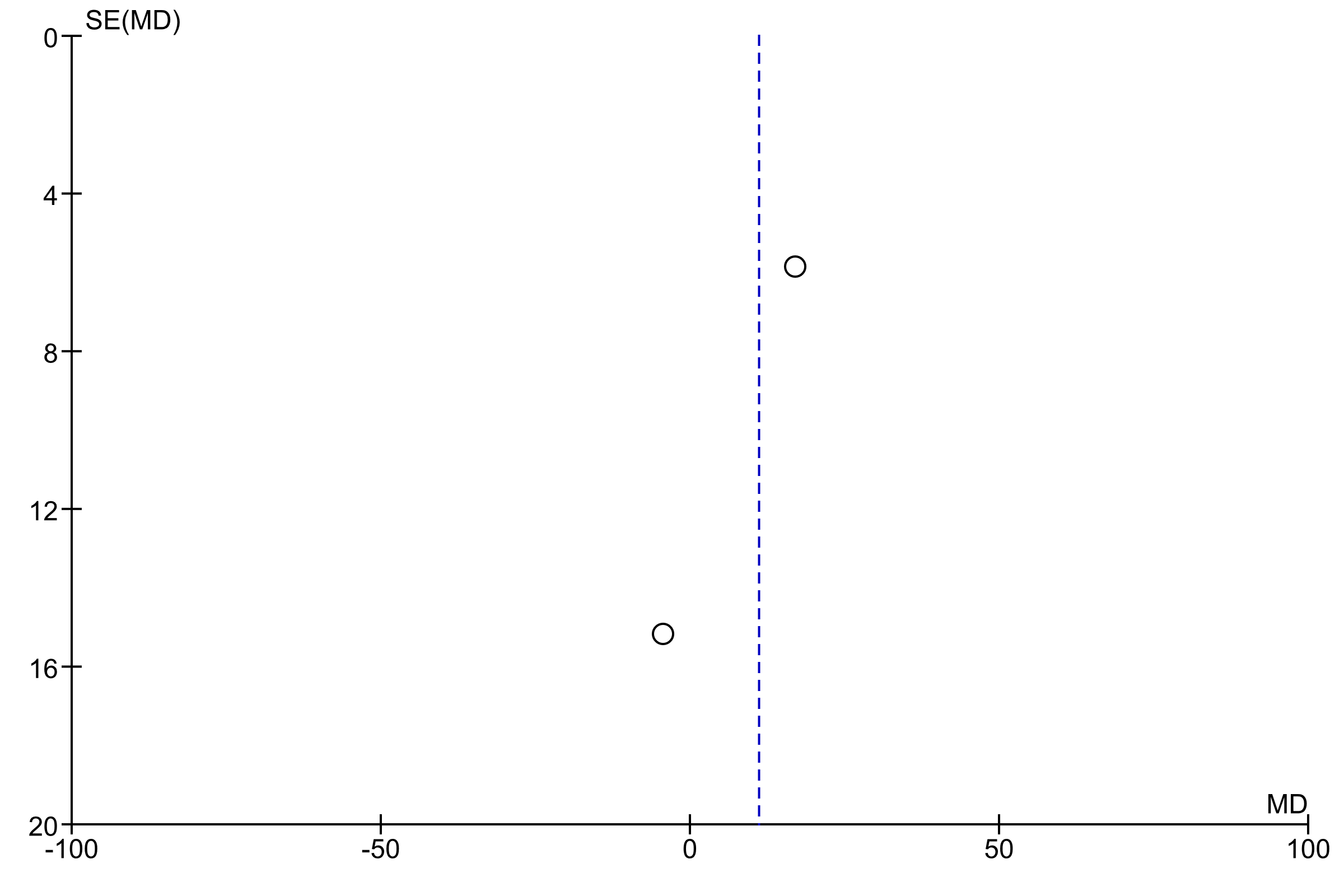


**Supplementary Figure 15** Funnel Plot for Trait Anxiety


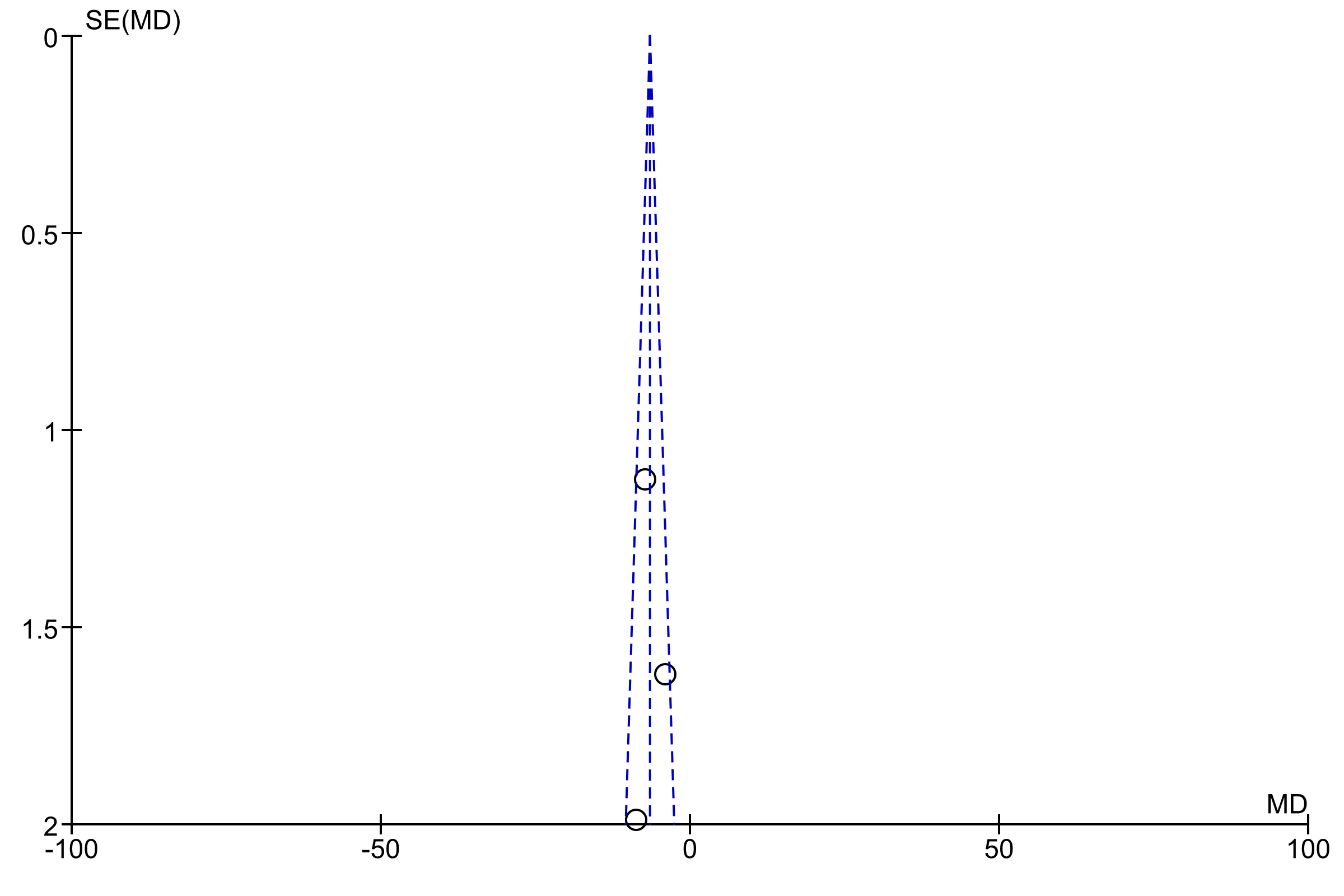


**Supplementary Figure 16** Funnel Plot for State Anxiety


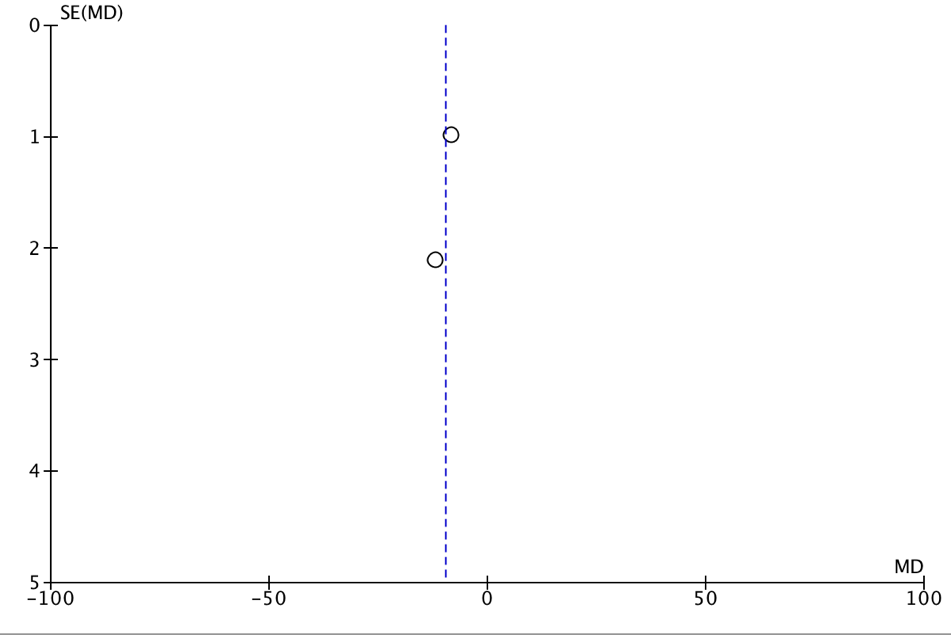


**Supplementary Figure 17** Funnel Plot for Depression


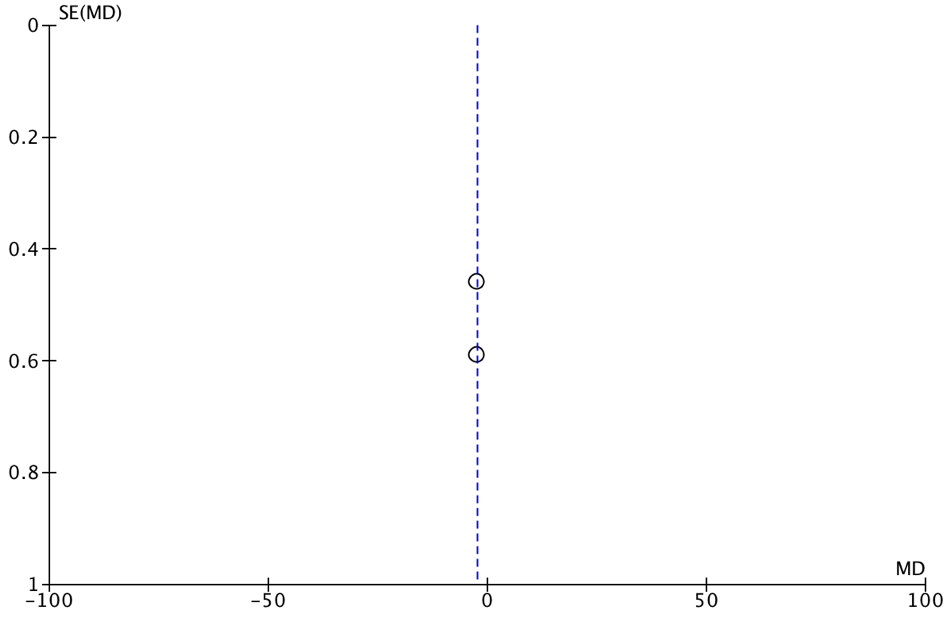


**Supplementary Table 1.** Search Strategies

| Medline, CINAHL, PsycInfo  October 10, 2023 |
| --- |
| 1. (MM “Endometriosis”)  2. (MM “Cognitive Behavioral Therapy”)  3. (MM “Behavior Therapy”)  4. (MM “Psychological Techniques”)  5. (MM “Psychology”)  6. (MM “Acceptance and Commitment Therapy”)  7. (MM “Behavioral Disciplines and Activities”)  8. (MM “Mental Health Services”)  9. (MM “Dialectical Behavior Therapy”)  10. (MM “Psychotherapy”)  11. TI “Endometriosis” OR “Pelvic Pain” OR “Chronic Pelvic Pain”  12. AB “Endometriosis” OR “Pelvic Pain” OR “Chronic Pelvic Pain”  13. TI “Psycholog*”, “Cognitive” OR “Behavioral” OR “Behavioural” OR “Mindfulness” OR “Relaxation”  14. AB “Psycholog*”, “Cognitive” OR “Behavioral” OR “Behavioural” OR “Mindfulness” OR “Relaxation”  15. TI “Intervention” OR “Therapy” OR “Treatment” OR “Psychotherapy” OR “Training”  16. AB “Intervention” OR “Therapy” OR “Treatment” OR “Psychotherapy” OR “Training”  17. S13 OR S14  18. S15 OR S16  19. S17 AND S18  20. S1 OR S11 OR S12  21. S2 OR S3 OR S4 OR S5 OR S6 OR S7 OR S8 OR S9 OR S10 OR S19  22. S20 AND S21 |
| CENTRAL  October 10, 2023 |
| 1. MeSH descriptor: [Endometriosis] explode all trees  2. MeSH descriptor: [Cognitive Behavioral Therapy] explode all trees  3. MeSH descriptor: [Behavior Therapy] explode all trees  4. MeSH descriptor: [Psychological Techniques] explode all trees  5. MeSH descriptor: [Psychology] explode all trees  6. MeSH descriptor: [Acceptance and Commitment Therapy] explode all trees  7. MeSH descriptor: [Behavioral Disciplines and Activities] explode all trees  8. MeSH descriptor: [Mental Health Services] explode all trees  9. MeSH descriptor: [Dialectical Behavior Therapy] explode all trees  10. MeSH descriptor: [Psychotherapy] explode all trees  11. (“Endometriosis” OR “Pelvic Pain” OR “Chronic Pelvic Pain”):ti,ab,kw (Word variations have been searched)  12. (“Psychological” OR “Cognitive” OR “Behavioral” OR “Behavioural” OR “Mindfulness” OR “Relaxation”):ti,ab,kw (Word variations have been searched)  13. (“Intervention” OR “Therapy” OR “Treatment” OR “Psychotherapy” OR “Training”):ti,ab,kw (Word variations have been searched)  14. #12 AND #13  15. #1 OR #11  16. #2 OR #3 OR #4 OR #5 OR #6 OR #7 OR #8 OR #9 OR #10 OR #14  17. #15 AND #16  18. Trials matching #17 |

**Supplementary Table 2.** Excluded Studies

| Abstract (N = 2) |
| --- |
| 1. Hansen, K., Kold, M., Kesmodel, U., & Forman, A. (2015). Long-time effects of mindfulness-based psychological treatment on chronic pain and quality of life in endometriosis. *Journal of Endometriosis and Pelvic Pain Disorders*, *7*(16), 1-82.<https://doi.org/10.5301/je.5000214> 2. Miazga, E., Starkman, H., Skolnik, E., Schroeder, N., Yarranton, B., Nensi, A., & McCaffrey, C. (2022). Virtual Mindfulness Therapy for the Management of Endometriosis Chronic Pelvic Pain: A Novel Delivery Platform to Increase Access to Care. *Journal of Minimally Invasive Gynecology*, *29*(11), S35.<https://doi.org/10.1016/j.jmig.2022.09.115> |
| Design (N = 14) |
| 1. Brief psychotherapy of a patient with headache and endometriosis. (1956). The American Journal of Medicine, 20(2), 286-291.<https://doi.org/10.1016/0002-9343(56)90197-8> 2. Champaneria, R., Daniels, J. P., Raza, A., Pattison, H. M., & Khan, K. S. (2012). Psychological therapies for chronic pelvic pain: Systematic review of randomized controlled trials. Acta Obstetricia et Gynecologica Scandinavica, 91(3), 281-286.<https://doi.org/10.1111/j.1600-0412.2011.01314.x> 3. Donatti, L., Malvezzi, H., Azevedo, B. C. D., Baracat, E. C., & Podgaec, S. (2022). Cognitive Behavioral Therapy in Endometriosis, Psychological Based Intervention: A Systematic Review. Revista Brasileira de Ginecologia e Obstetrícia / RBGO Gynecology and Obstetrics, 44(03), 295-303.<https://doi.org/10.1055/s-0042-1742406> 4. Evans, S., Fernandez, S., Olive, L., Payne, L. A., & Mikocka-Walus, A. (2019). Psychological and mind-body interventions for endometriosis: A systematic review. Journal of Psychosomatic Research, 124, 109756.<https://doi.org/10.1016/j.jpsychores.2019.109756> 5. Friggi Sebe Petrelluzzi, K., Garcia, M. C., Petta, C. A., Ribeiro, D. A., De Oliveira Monteiro, N. R., Céspedes, I. C., & Spadari, R. C. (2012). Physical therapy and psychological intervention normalize cortisol levels and improve vitality in women with endometriosis. Journal of Psychosomatic Obstetrics & Gynecology, 33(4), 191-198.<https://doi.org/10.3109/0167482X.2012.729625> 6. Hansen, K. E., Kesmodel, U. S., Kold, M., & Forman, A. (2017). Long-term effects of mindfulness-based psychological intervention for coping with pain in endometriosis: A six-year follow-up on a pilot study. Nordic Psychology, 69(2), 100-109.<https://doi.org/10.1080/19012276.2016.1181562> 7. Kames, L. D., Rapkin, A. J., Naliboff, B. D., Afifi, S., & Ferrer-Brechner, T. (1990). Effectiveness of an interdisciplinary pain management program for the treatment of chronic pelvic pain. Pain, 41(1), 41-46.<https://doi.org/10.1016/0304-3959(90)91107-T> 8. Kistner, R. (1965). Dysmenorrhea and endometriosis. Modern treatment, 2, 150-164. 9. Kold, M., Hansen, T., Vedsted-Hansen, H., & Forman, A. (2012). Mindfulness-based psychological intervention for coping with pain in endometriosis. Nordic Psychology, 64(1), 2-16.<https://doi.org/10.1080/19012276.2012.693727> 10. Matloobi, M., Amini, L., Shahali, S., Haghani, H., Tahermanesh, K., Hassanlouei, B., Allahqoli, L., & Alkatout, I. (2022). Effect of sex education on sexual function and sexual quality of life in women with endometriosis: A quasi‐experimental study. International Journal of Gynecology & Obstetrics, 159(3), 702-710.<https://doi.org/10.1002/ijgo.14254> 11. Rogers, S. K., Galloway, A., Hirsh, A. T., Zapolski, T., Chen, C. X., & Rand, K. L. (2023). Efficacy of psychological interventions for dysmenorrhea: A meta-analysis. Pain Medicine, 24(9), 1086-1099.<https://doi.org/10.1093/pm/pnad058> 12. Samami, E., Shahhosseini, Z., Khani, S., & Elyasi, F. (2023). Pain‐focused psychological interventions in women with endometriosis: A systematic review. Neuropsychopharmacology Reports, 43(3), 310-319.<https://doi.org/10.1002/npr2.12348> 13. Van Niekerk, L., Weaver-Pirie, B., & Matthewson, M. (2019). Psychological interventions for endometriosis-related symptoms: A systematic review with narrative data synthesis. Archives of Women’s Mental Health, 22(6), 723-735.<https://doi.org/10.1007/s00737-019-00972-6> 14. Wu, S., Wang, X., Liu, H., & Zheng, W. (2022). Efficacy of cognitive behavioral therapy after the surgical treatment of women with endometriosis: A preliminary case-control study. Medicine, 101(51), e32433.<https://doi.org/10.1097/MD.0000000000032433> |
| Patient (N = 5) |
| 1. Ariza-Mateos, M. J., Cabrera-Martos, I., López-López, L., Rodríguez-Torres, J., Torres-Sánchez, I., & Valenza, M. C. (2020). Effects of a patient-centered program including the cumulative-complexity model in women with chronic pelvic pain: A randomized controlled trial. Maturitas, 137, 18-23.<https://doi.org/10.1016/j.maturitas.2020.04.005> 2. Crisp, C. D., Baldi, R., Fuller, M., Abreu, E., & Nackley, A. G. (2023). Complementary Approaches for Military Women with Chronic Pelvic Pain: A Randomized Trial. Journal of Integrative and Complementary Medicine, 29(1), 22-30.<https://doi.org/10.1089/jicm.2022.0616> 3. Forbes, G., Newton, S., Cantalapiedra Calvete, C., Birch, J., Dodds, J., Steed, L., Rivas, C., Khan, K., Röhricht, F., Taylor, S., Kahan, B. C., & Ball, E. (2020). MEMPHIS: A smartphone app using psychological approaches for women with chronic pelvic pain presenting to gynaecology clinics: a andomized feasibility trial. BMJ Open, 10(3), e030164.<https://doi.org/10.1136/bmjopen-2019-030164> 4. Norman, S. A., Lumley, M. A., Dooley, J. A., & Diamond, M. P. (2004). For Whom Does It Work? Moderators of the Effects of Written Emotional Disclosure in a Randomized Trial Among Women With Chronic Pelvic Pain. Psychosomatic Medicine, 66(2), 174-183.<https://doi.org/10.1097/01.psy.0000116979.77753.74> 5. Zarski, A.-C., Berking, M., & Ebert, D. D. (2021). Efficacy of internet-based treatment for genito-pelvic pain/penetration disorder: Results of a randomized controlled trial. Journal of Consulting and Clinical Psychology, 89(11), 909-924.<https://doi.org/10.1037/ccp0000665> |
| Outcome (N = 3) |
| 1. Beissner, F., Preibisch, C., Schweizer-Arau, A., Popovici, R. M., & Meissner, K. (2018). Psychotherapy With Somatosensory Stimulation for Endometriosis-Associated Pain: The Role of the Anterior Hippocampus. Biological Psychiatry, 84(10), 734-742.<https://doi.org/10.1016/j.biopsych.2017.01.006> 2. Moreira, M. F., Gamboa, O. L., & Oliveira, M. A. P. (2024). Mindfulness-Based Intervention Effect on the Psychophysiological Marker of Self-Regulation in Women With Endometriosis-Related Chronic Pain. The Journal of Pain, 25(1), 118-131.<https://doi.org/10.1016/j.jpain.2023.07.026> 3. Sherman, K. A., Pehlivan, M. J., Redfern, J., Armour, M., Dear, B., Singleton, A., Duckworth, T., Ciccia, D., Cooper, M., Hawkey, A., Parry, K. A., & Gandhi, E. (2023). A supportive text message intervention for individuals living with endometriosis (EndoSMS): Randomized controlled pilot and feasibility trial. Contemporary Clinical Trials Communications, 32, 101093.<https://doi.org/10.1016/j.conctc.2023.101093> |
| Language (N = 2) |
| 1. Begovich, E., Solopova, A. G., Khlopkova, S. V., Son, E. A., Ungiadze, J. Yu., & Idrisova, L. E. (2022). Assessing sexual dysfunction in patients with external genital endometriosis. Obstetrics, Gynecology and Reproduction, 16(4), 354-364.<https://doi.org/10.17749/2313-7347/ob.gyn.rep.2022.345> 2. Jun, Z., & Yanling, A. (2015). Influence of cognitive behavior therapy on psychological and quality of life of patients with endometriosis. Chinese Nursing Research, 30(5), 1740-1743.<https://doi.org/10.3969/j.issn.1009-6493.2016.14.026> |
| Protocol (N = 13) |
| 1. Ball, E. (2016). Mindfulness Meditation for Chronic Pelvic Pain Management. Número de identificación del protocolo: NCT02721108. <https://www.cochranelibrary.com/central/doi/10.1002/central/CN-01556883/full> 2. Behboodi Moghadam, Z. (2021). The effect of education on promoting sexual health and fertility in women with endometriosis. Número de identificación del protocolo: IRCT20120414009463N64. <https://www.cochranelibrary.com/central/doi/10.1002/central/CN-02281269/full> 3. Bruenahl, C. A. (2016). Pilot study for the evaluation of a combined psycho- and physiotherapeutic treatment program for patients with chronic pelvic pain syndrome (CPPS). Número de identificación del protocolo: ISRCTN43221600. <https://www.cochranelibrary.com/central/doi/10.1002/central/CN-02444650/full> 4. Chaichian, S. (2018). Efficacy of sexual counseling on sexual behaviors in women with endometriosis, candidates for laparoscopic surgery. Número de identificación del protocolo: IRCT20180130038567N1. <https://www.cochranelibrary.com/central/doi/10.1002/central/CN-01904848/full> 5. Hansen, K.E., Vase, L. & Forman, A. (2016). Psychological Pain Treatment in Endometriosis. Número de identificación del protocolo: NCT02761382. <https://www.cochranelibrary.com/central/doi/10.1002/central/CN-01557948/full> 6. Kadah, S. (2022). Pelvic floor muscle exercises and mindfulness for women with endometriosis-associated pelvic pain. Número de identificación del protocolo: ACTRN12622001393741. <https://www.cochranelibrary.com/central/doi/10.1002/central/CN-02493724/full> 7. Mikocka-Walus, A., Druitt, M., O’Shea, M., Skvarc, D., Watts, J. J., Esterman, A., Tsaltas, J., Knowles, S., Harris, J., Dowding, C., Parigi, E., & Evans, S. (2021). Yoga, cognitive–behavioural therapy versus education to improve quality of life and reduce healthcare costs in people with endometriosis: A randomized controlled trial. BMJ Open, 11(8), e046603.<https://doi.org/10.1136/bmjopen-2020-046603> 8. Moreira, M. F. (2019). Impact of Mindfulness Meditation Program on pain and quality of life in women with deep Endometriosis. Número de identificación del protocolo: RBR-65b5b3. <https://www.cochranelibrary.com/central/doi/10.1002/central/CN-02071384/full> 9. Nap, W. (2020). Cognitive Behavioral Therapy in Endometriosis. Número de identificación del protocolo: NCT04448366. <https://www.cochranelibrary.com/central/doi/10.1002/central/CN-02129688/full> 10. Nikseresht, M. & Erfanian, F. (2021). The effect of cognitive-behavioral group counseling on reproductive health and sexual satisfaction in women with endometriosis. Número de identificación del protocolo: IRCT20210919052516N1. [https://www.cochranelibrary.com/central/doi/10.1002/central/CN-02351560/ful](https://www.cochranelibrary.com/central/doi/10.1002/central/CN-02351560/full)l 11. Parigi, E. & Evans, S. (2020). Evaluating the influence of Yoga, Cognitive Behaviour Therapy and Standard Care on Quality of Life and Healthcare Costs in Endometriosis. Número de identificación del protocolo: ACTRN12620000756921. <https://www.cochranelibrary.com/central/doi/10.1002/central/CN-02184347/full> 12. Tarus, H.A. & Demirci,N. (2023) The Effect of the Self-Care Support Program on Women With Endometriosis. Número de identificación del protocolo: NCT05861739. <https://www.cochranelibrary.com/central/doi/10.1002/central/CN-02561476/full> 13. Weise, C. (2021). Efficacy of Internet-based Cognitive Behavioral Therapy for Endometriosis (EdiTh). Número de identificación del protocolo: NCT05098444. <https://www.cochranelibrary.com/central/doi/10.1002/central/CN-02341357/full> |

**Supplementary Table 3.** Summary of Findings of Psychological Interventions Compared to Standard Care for the Treatment of Endometriosis

| **Anticipated absolute effects‡ (95% CI)** | | | | |
| --- | --- | --- | --- | --- |
| **Outcomes** | **Risk with control** | **Risk with psychological intervention** | **N  (studies)** | **Certainty of the evidence  (GRADE)** |
| **PAIN** | | | | |
| Dyspareunia assessed with: NRS and VAS follow-up: range 1 week to 3 months | - | SMD **0.54 lower** (0.86 lower to 0.22 lower) | 160 (3 RCTs)^1,2,3^ | ⨁⨁⨁◯ Moderate^a^ |
| Dyschezia assessed with: NRS follow-up: range 1 week to 3 months | The mean was **5.63** | MD **2.9 lower** (4.55 lower to 1.26 lower) | 88 (2 RCTs)^2,3^ | ⨁⨁⨁◯ Moderate^a^ |
| Pelvic pain assessed with: NRS follow-up: range 1 week to 3 months | The mean was **8.02** | MD **1.22 lower** (2.23 lower to 0.22 lower) | 107 (2 RCTs)^2,3^ | ⨁⨁⨁◯ Moderate^a^ |
| **QUALITY OF LIFE** | | | | |
| General health assessed with: SF-36 follow-up: range 1 week to 3 months | In the study by Moreira et al. (2022), no improvement was observed in the brief mindfulness-based intervention group compared to the standard medical care (MD: 4.50, 95% CI: -0.06, 9.06; N = 54). In contrast, in the Zhao et al. study (2012), statistically significant differences were identified in the scores of the progressive muscle relaxation training compared to the group that did not receive it (MD: -20.54, 95% CI: -28.48, -12.60; N = 87). | | 141 (2 RCTs)^2,4^ | ⨁◯◯◯ Very low^b,c^ |
| Vitality assessed with: SF-36 follow-up: range 1 week to 3 months | Moreira et al. (2022) reported an improvement in favor of the brief mindfulness-based intervention compared to the standard medical care group (MD: -6.25, 95% CI: -13.90, 1.40; N = 54), whereas Zhao et al. (2012) did not report a significant difference between progressive muscle relaxation training and routine care with gonadotropin- releasing hormone analogues (MD: 14.67, 95% CI: 6.33, 23.01; N = 87). | | 141 (2 RCTs)^2,4^ | ⨁◯◯◯ Very low^b,c^ |
| Social function assessed with: SF-36 follow-up: range 1 week to 3 months | The mean was **45.96** | MD **4.47 lower** (26.29 lower to 17.35 higher) | 141 (2 RCTs)^2,4^ | ⨁⨁◯◯ Low^a,d,e^ |
| Emotional role assessed with: SF-36 follow-up: range 1 week to 3 months | The mean was **19.27** | MD **15.98 lower** (35.22 lower to 3.27 higher) | 141 (2 RCTs)^2,4^ | ⨁⨁◯◯ Low^a,e,f^ |
| Mental health assessed with: SF-12 and SF-36 follow-up: range 1 week to 3 months | - | SMD **1.13 higher** (0.3 higher to 1.96 higher) | 277 (4 RCTs)^2,3,4,5^ | ⨁⨁⨁◯ Moderate^a,g^ |
| Physical functioning assessed with: SF-36 follow-up: range 1 week to 3 months | The mean was **56.01** | MD **8.11 higher** (5.61 lower to 21.83 higher) | 141 (2 RCTs)^2,4^ | ⨁⨁◯◯ Low^a,e,f^ |
| Physical role assessed with: SF-36 follow-up: range 1 week to 3 months | The mean was **23.40** | MD **10.98 higher** (7.52 lower to 29.49 higher) | 141 (2 RCTs)^2,4^ | ⨁⨁⨁◯ Moderate^a,e^ |
| Physical health assessed with: SF-12 and SF-36 follow-up: range 1 month to 3 months | In both studies, a significant difference in favor of the group receiving psychological intervention compared to the control was identified, which was higher in Farshi et al. study (2020) (SMD: 3.72, 95% CI: 2.97, 4.48; N = 76), than in Meissner et al. study (2016) (SMD: 0.60, 95% CI: 0.08, 1.12; N = 60). | | 136 (2 RCTs)^3,5^ | ⨁◯◯◯ Very low^a,b^ |
| **ANXIETY** | | | | |
| Trait anxiety assessed with: STAI follow-up: range 1 month to 3 months | The mean was **46.25** | MD **6.63 lower** (8.27 lower to 4.99 lower) | 216 (3 RCTs)^3,4,5^ | ⨁⨁⨁⨁ Moderate^a^ |
| State anxiety assessed with: STAI follow-up: mean 3 months | The mean was **44.39** | MD **9.72 lower** (13.11 lower to 6.33 lower) | 163 (2 RCTs)^4,5^ | ⨁⨁◯◯ Low^a,f^ |
| **DEPRESSION** | | | | |
| Depression assessed with: HADS follow-up: mean 3 months | The mean was **6.46** | MD **2.49 lower** (3.2 lower to 1.79 lower) | 144 (2 RCTs)^3,4^ | ⨁⨁⨁◯ Moderate^a,h^ |
| **‡The risk in the intervention group** (and its 95% confidence interval) is based on the assumed risk in the comparison group and the **relative effect** of the intervention (and its 95% CI). **CI:** confidence interval; **HADS**: Hospital Anxiety and Depression Scale; **N:** number of patients; **STAI:** State-trait Anxiety Inventory; **MD:** mean difference; **NRS:** 0–10-point numeric rating scale; **SF-36:** Short Form-36 Health Survey; **SMD:** standardized mean difference | | | | |
| **GRADE Working Group grades of evidence High certainty:** we are very confident that the true effect lies close to that of the estimate of the effect. **Moderate certainty:** we are moderately confident in the effect estimate: the true effect is likely to be close to the estimate of the effect, but there is a possibility that it is substantially different. **Low certainty:** our confidence in the effect estimate is limited: the true effect may be substantially different from the estimate of the effect. **Very low certainty:** we have very little confidence in the effect estimate: the true effect is likely to be substantially different from the estimate of effect. | | | | |

#### Explanations

a. Small sample size (N= <400).

b. The analysis showed the presence of high and unexplained heterogeneity rates (I^2^= >90%), so the pooled data are not presented.

c. Inconsistent effects of the intervention across studies.

d. The analysis showed substantial and unexplained heterogeneity rates (I^2^= >75%).

e. Wide confidence interval.

f. The analysis showed substantial and unexplained heterogeneity rates (I^2^= >50%).

g. High heterogeneity was detected (I^2^= 90%) however, sensitivity analysis revealed that Farshi et al. study (2020) contributed to the entirety of the heterogeneity. Nevertheless, even after excluding this study, a moderate effect in favor of psychological intervention over waitlist or usual treatment was maintained (SMD: 0.70, 95% CI: 0.42, 0.99; I^2^= 0%; N= 201; K= 3).

h. In the initial analysis, high heterogeneity was detected (I^2^= 90%). In the sensitivity analysis conducted, it was found that the study by Farshi et al. (2020) was the source of all the heterogeneity, so it was excluded.

#### References

1. Tajik, M., Shahali, S., & Shadjoo, K. (2022). The co-effect of sensate focus technique and sexual position changing on sexual function of women who use medical treatment for endometriosis. *Journal of Obstetrics and Gynaecology*, *42*(8), 3706-3711.<https://doi.org/10.1080/01443615.2022.2158316>

2. Moreira, M. D. F., Gamboa, O. L., & Pinho Oliveira, M. A. (2022). A single‐blind, randomized, pilot study of a brief mindfulness‐based intervention for the endometriosis‐related pain management. European Journal of Pain, 26(5), 1147-1162. https://doi.org/10.1002/ejp.1939

3. Meissner, K., Schweizer-Arau, A., Limmer, A., Preibisch, C., Popovici, R. M., Lange, I., De Oriol, B., & Beissner, F. (2016). Psychotherapy With Somatosensory Stimulation for Endometriosis-Associated Pain: A Randomized Controlled Trial. *Obstetrics & Gynecology*, *128*(5), 1134-1142.<https://doi.org/10.1097/AOG.0000000000001691>

4. Zhao, L., Wu, H., Zhou, X., Wang, Q., Zhu, W., & Chen, J. (2012). Effects of progressive muscular relaxation training on anxiety, depression and quality of life of endometriosis patients under gonadotrophin-releasing hormone agonist therapy. *European Journal of Obstetrics & Gynecology and Reproductive Biology*, *162*(2), 211-215.<https://doi.org/10.1016/j.ejogrb.2012.02.029>

5. Farshi, N., Hasanpour, S., Mirghafourvand, M., & Esmaeilpour, K. (2020). Effect of self-care counselling on depression and anxiety in women with endometriosis: A randomized controlled trial. BMC Psychiatry, 20(1), 391. <https://doi.org/10.1186/s12888-020-02795-7>

**Supplementary Table 4.** Main Data Extracted from the Included Studies: Pain

| First author, year | Comparison | Outcomes | Follow-up | Treatment | | | Control | | | P- value |
| --- | --- | --- | --- | --- | --- | --- | --- | --- | --- | --- |
|  |  |  |  | Mean | SD/IC95% | N | Mean | SD/IC95% | N |  |
| Hansen et al., 2023 | MYENDO+ non-specific psychological intervention vs. waiting list control (usual care) | NRS: Pain intensity | Postintervention | NI | NI | 38 | NI | NI | 16 | 0.009 |
|  |  | NRS: Pain unpleasantness |  | NI | NI | 38 | NI | NI | 16 | 0.009 |
|  |  | PPT: Pressure pain detection threshold |  | 34.04 | 15.81 | 38 | 53.79 | 25.77 | 16 | 0.562 |
|  | MYENDO vs. non-specific psychological intervention | NRS: Pain intensity |  | NI | NI | 19 | NI | NI | 19 | 0.037 |
|  |  | NRS: Pain unpleasantness |  | NI | NI | 19 | NI | NI | 19 | 0.049 |
|  |  | PPT: Pressure pain detection threshold |  | 36.61 | 21.6 | 19 | 31.48 | 7.36 | 19 | 0.615 |
| Meissner et al., 2016 | Psychotherapy  with somatosensory stimulation vs. waitlist  control | NRS: Maximum overall pain | 3 months | -3 | (-3.8 to -2.1) | 32 | -0.9 | (-1.8 to 0.1) | 28 | 0.002 |
|  |  | NRS: Average overall pain |  | -2.5 | (-3.2 to -1.7) | 31 | 0 | (-0.8 to 0.7) | 28 | <0.001 |
|  |  | NRS: Maximum pelvic pain |  | -2.4 | (-3.3 to -1.5) | 27 | -1 | (-1.9 to -0.1) | 26 | 0.036 |
|  |  | NRS: Maximum dyschezia |  | -3.1 | (-4.9 to -1.3) | 13 | 0.4 | (-0.9 to 1.8) | 21 | 0.003 |
|  |  | NRS: Maximum dyspareunia |  | -2.9 | (-5 to -0.9) | 9 | -1.1 | (-2.6 to 0.4) | 17 | 0.15 |
|  |  | NRS: Maximum overall pain | 6 months | -2.9 | (-3.9 to -1.9) | 20 | -3.7 | (-4.7 to -2.7) | 20 | 0.261 |
|  |  | NRS: Average overalll pain |  | -2.6 | (-3.6 to -1.5) | 19 | -2.3 | (-3.3 to -1.2) | 18 | 0.683 |
|  |  | NRS: Maximum pelvic pain |  | -3.4 | (-4.7 to -2.2) | 20 | -3.3 | (-4.6 to -1.9) | 18 | 0.823 |
|  |  | NRS: Maximum dyschezia |  | -4.8 | (-7.0 to -2.8) | 8 | -2 | (-3.7 to -0.3) | 12 | 0.04 |
|  |  | NRS: Maximum dyspareunia |  | -1.9 | (-4.3 to 0.5) | 4 | -2.7 | (-4.1 to -1.2) | 11 | 0.548 |
|  |  | NRS: Maximum overall pain | 24 months | -4.1 | (-5.3 to -2.9) | 30 | -3 | (-4.3 to -1.7) | 26 | 0.227 |
|  |  | NRS: Average overall pain |  | -3.3 | (-4.4 to -2.1) | 28 | -1.3 | (-2.5 to -0.1) | 26 | 0.019 |
|  |  | NRS: Maximum pelvic pain |  | -3.8 | (-5.0 to -2.6) | 29 | -2.7 | (-4 to -1.4) | 26 | 0.229 |
|  |  | NRS: Maximum dyschezia |  | -4 | (-5.7 to -2.2) | 12 | -1.3 | (-2.7 to 0.0) | 20 | 0.02 |
|  |  | NRS: Maximum dyspareunia |  | -4.8 | (-7.4 to -2.2) | 10 | -2.6 | (-4.7 to -0.5) | 15 | 0.175 |
| Moreira et al., 2022 | Brief Mindfulness-Based  Intervention vs. Standard  medical care | NRS: Pelvic pain | Postintervention 1 week | 5.33^†^ | 3.7^†^ | 26 | 6.17^†^ | 2.96^†^ | 28 | 0.013 |
|  |  | NRS: Pain unpleasantness |  | 4.42^†^ | 3.52^†^ | 26 | 6.58^†^ | 3.15^†^ | 28 | 0 |
|  |  | NRS: Dysuria |  | 2.58^†^ | 4.26^†^ | 26 | 2.58^†^ | 3.15^†^ | 28 | 0.604 |
|  |  | NRS: Dyspareunia |  | 2^†^ | 3.7^†^ | 26 | 4.75^†^ | 4.63^†^ | 28 | 0.355 |
|  |  | NRS: Dyschezia |  | 3^†^ | 5.19^†^ | 26 | 4.75^†^ | 5.37^†^ | 28 | 0.007 |
|  |  | NRS: Dysmenorrhoea |  | 3.75^†^ | 5.74^†^ | 26 | 7^†^ | 4.07^†^ | 28 | 0.286 |
|  |  | NRS: Pelvic pain | 4 weeks | 4.42^†^ | 2.78^†^ | 22 | 5.83^†^ | -2.22^†^ | 24 | 0.007 |
|  |  | NRS: Pain unpleasantness |  | 3.5^†^ | 3.7^†^ | 22 | 5.67^†^ | 2.22^†^ | 24 | 0.004 |
|  |  | NRS: Dysuria |  | 2^†^ | 2.96^†^ | 22 | 2.92^†^ | 3.15^†^ | 24 | 0.033 |
|  |  | NRS: Dyspareunia |  | 0.92^†^ | 2.04^†^ | 22 | 4.08^†^ | 3.15^†^ | 24 | 0 |
|  |  | NRS: Dyschezia |  | 1.83^†^ | 2.96^†^ | 22 | 4.08^†^ | 4.63^†^ | 24 | 0.002 |
|  |  | NRS: Dysmenorrhoea |  | 2.08^†^ | 3.52^†^ | 22 | 5.92^†^ | 3.89^†^ | 24 | 0.016 |
| Tajik et al., 2022 | Sensate focus technique and sexual positions vs. routine care | VAS: sexual pain | Postintervention 1 month | 3.53 | 2.16 | 40 | 4.63 | 2.59 | 40 | 0.04 |
|  |  | VAS: sexual pain | 2 months | 2.7 | 1.71 | 40 | 5 | 2.61 | 40 | <0.001 |
| Note. NRS: 0–10-point numeric rating scale; N: number of patients; SD: standard deviation; VAS: Visual analogue scale  ^†^ own calculation | | | | | | | | | | |

**Supplementary Table 5.** Main Data Extracted from the Included Studies: Quality of Life

| First author, year | Comparison | Outcomes | Follow-up | Treatment | | | Control | | | P- value |
| --- | --- | --- | --- | --- | --- | --- | --- | --- | --- | --- |
|  |  |  |  | Mean | SD/IC95% | N | Mean | SD/IC95% | N |  |
| Farshi et al., 2020 | Self-care group counselling vs. Routine care | SF-36: Physical health | Postintervention  4 weeks | 58.9 | 4.9 | 38 | 39.5 | 5.4 | 38 | <0.001 |
|  |  | SF-36: Mental health |  | 57.1 | 4.8 | 38 | 44.7 | 4.8 | 38 | <0.001 |
| Hansen et al., 2023 | MYENDO+ non-specific psychological intervention vs. waiting list control (usual care) | EHP-30 QoL: Pain | Postintervention | 42.61 | 19.77 | 38 | 48.78 | 12.61 | 16 | 0.180 |
|  |  | EHP-30 QoL: Control and powerlessness |  | 48.21 | 18.3 | 38 | 60.26 | 20.53 | 16 | 0.019 |
|  |  | EHP-30 QoL: Emotional wellbeing |  | 33.62 | 11.51 | 38 | 48.71 | 22.66 | 16 | 0.003 |
|  |  | EHP-30 QoL: Social support |  | 37.07 | 21.52 | 38 | 53.37 | 27.79 | 16 | 0.042 |
|  |  | EHP-30 QoL: Self-image |  | 44.54 | 24.43 | 38 | 48.07 | 30.08 | 16 | 0.312 |
|  | MYENDO vs. non-specific psychological intervention | EHP-30 QoL: Pain |  | 42.31 | 21.87 | 19 | 42.88 | 18.54 | 19 | 0.586 |
|  |  | EHP-30 QoL: Control and powerlessness |  | 50 | 19.17 | 19 | 46.67 | 18.04 | 19 | 0.801 |
|  |  | EHP-30 QoL: Emotional wellbeing |  | 39.29 | 11.52 | 19 | 28.33 | 8.94 | 19 | 0.165 |
|  |  | EHP-30 QoL: Social support |  | 43.75 | 21.09 | 19 | 30.83 | 20.66 | 19 | 0.136 |
|  |  | EHP-30 QoL: Self-image |  | 40.48 | 28.09 | 19 | 48.33 | 20.7 | 19 | 0.631 |
| Meissner et al., 2016 | Psychotherapy  with somatosensory stimulation vs. waiting list  control | SF-12: Mental health | 3 months | 5,1 | (1.4 to 8.8) | 32 | -0,8 | (-4.8 to 3.1) | 28 | 0.031 |
|  |  | SF-12: Physical health | 6 months | 5.9 | (3.0 to 8.7) | 21 | 7,4 | (4.5 to 10.3) | 20 | 0.446 |
|  |  | SF-12: Mental health |  | 6.4 | (1.5–11.4) | 21 | 2,9 | (-2.2 to 8) | 20 | 0.329 |
|  |  | SF-12: Physical health | 24 months | NR | NR | NR | NR | NR | NR | NR |
|  |  | SF-12: Physical health |  | NR | NR | NR | NR | NR | NR | NR |
| Moreira et al., 2022 | Brief Mindfulness-Based  Intervention + vs. Standard  medical care | SF-36: Physical summary | Postintervention  1 week | 185^†^ | 91.11^†^ | 26 | 178.5^†^ | 100.37^†^ | 28 | 0.135 |
|  |  | SF-36: Mental summary |  | 188.8^†^ | 113.64^†^ | 26 | 163.26^†^ | 85.46^†^ | 28 | 0.13 |
|  |  | SF-36: Physical functioning |  | 54.58^†^ | 30.56^†^ | 26 | 56.25^†^ | 36.11^†^ | 28 | 0.245 |
|  |  | SF-36: Physical role |  | 29.17^†^ | 55.56^†^ | 26 | 33.33^†^ | 55.56^†^ | 28 | 0.102 |
|  |  | SF-36: Bodily pain |  | 45.42^†^ | 21.3^†^ | 26 | 43.75^†^ | 25^†^ | 28 | 0.062 |
|  |  | SF-36: General health |  | 49.5^†^ | 7.41^†^ | 26 | 54^†^ | 9.63^†^ | 28 | 0.62 |
|  |  | SF-36: Vitality |  | 50.42^†^ | 13.89^†^ | 26 | 44.17^†^ | 14.81^†^ | 28 | 0.097 |
|  |  | SF-36: Social function |  | 53.13^†^ | 25.46^†^ | 26 | 45.83^†^ | 27.78^†^ | 28 | 0.465 |
|  |  | SF-36: Emotional role |  | 22.22^†^ | 49.39^†^ | 26 | 25^†^ | 30.87^†^ | 28 | 0.144 |
|  |  | SF-36: Mental health |  | 54.33^†^ | 28.89^†^ | 26 | 41^†^ | 17.04^†^ | 28 | 0.007 |
|  |  | SF-36: Physical summary | 1 week | 198.08^†^ | 102.04^†^ | 22 | 174.33^†^ | 100^†^ | 24 | 0.118 |
|  |  | SF-36: Mental summary |  | 179.75^†^ | 121.6^†^ | 22 | 169.96^†^ | 85.21^†^ | 24 | 0.298 |
|  |  | SF-36: Physical functioning |  | 55^†^ | 33.33^†^ | 22 | 61.25^†^ | 26.85^†^ | 24 | 0.113 |
|  |  | SF-36: Physical role |  | 33.33^†^ | 55.56^†^ | 22 | 29.17^†^ | 55.56^†^ | 24 | 0.106 |
|  |  | SF-36: Bodily pain |  | 55.5^†^ | 24.07^†^ | 22 | 47.92^†^ | 15.74^†^ | 24 | 0.061 |
|  |  | SF-36: General health |  | 42.25^†^ | 22.78^†^ | 22 | 36^†^ | 21.85^†^ | 24 | 0.232 |
|  |  | SF-36: Vitality |  | 51.25^†^ | 13.89^†^ | 22 | 42.5^†^ | 11.11^†^ | 24 | 0.01 |
|  |  | SF-36: Social function |  | 48.96^†^ | 25.47^†^ | 22 | 51.04^†^ | 30.09^†^ | 24 | 0.558 |
|  |  | SF-36: Emotional role |  | 27.77^†^ | 49.38^†^ | 22 | 33.33^†^ | 49.39^†^ | 24 | 0.145 |
|  |  | SF-36: Mental health |  | 50.33^†^ | 31.85^†^ | 22 | 37.67^†^ | 22.96^†^ | 24 | 0.002 |
| Zandi et al., 2023 | Education based on the theory of planned behavior vs. Routine hospital care | ERHQ: Reproductive health | Postintervention  4 weeks | 124.91 | 8.9 | 35 | 84.65 | 11.14 | 36 | <0.001 |
|  |  | ERHQ: Reproductive health | 8 weeks | 125.22 | 9.69 | 35 | 82.62 | 18.55 | 36 | <0.001 |
| Zhao et al., 2012 | Progressive muscle relaxation training + GnRH vs. GnRH | SF-36: Quality of life | Postintervention | 83.45 | 20.46 | 42 | 68.67 | 18.49 | 45 | <0.001 |
|  |  | SF-36: Physical functioning |  | 88.59 | 14.31 | 42 | 75.47 | 17.83 | 45 | <0.001 |
|  |  | SF-36: Physical role |  | 72.13 | 27.84 | 42 | 55.26 | 26.75 | 45 | 0.008 |
|  |  | SF-36: Bodily pain |  | 69.86 | 17.41 | 42 | 49.38 | 22.52 | 45 | <0.001 |
|  |  | SF-36: General health |  | 87.78 | 16.53 | 42 | 67.24 | 21.12 | 45 | <0.001 |
|  |  | SF-36: Vitality |  | 59.96 | 20.44 | 42 | 74.63 | 19.16 | 45 | <0.001 |
|  |  | SF-36: Social function |  | 68.37 | 25.09 | 42 | 83.37 | 21.14 | 45 | 0.021 |
|  |  | SF-36: Emotional role |  | 4264 | 11.32 | 42 | 65.92 | 13.45 | 45 | <0.001 |
|  |  | SF-36: Mental health |  | 77.94 | 15.11 | 42 | 61.35 | 20.79 | 45 | <0.001 |

Note. EHP-30 QoL: Danish version of The Endometriosis Health Profile 30 questionnaire; N: number of patients; SD: standard deviation; SF-36: Short Form-36 Health Survey; SF-12: Short Form-12 Health Survey

^†^ own calculation

**Supplementary Table 6.** Main Data Extracted from the Included Studies: Anxiety

| First author, year | Comparison | Scale/Outcomes | Follow-up | Treatment | | | Control | | | P- value |
| --- | --- | --- | --- | --- | --- | --- | --- | --- | --- | --- |
|  |  |  |  | Mean | SD/IC95% | N | Mean | SD/IC95% | N |  |
| Farshi et al., 2020 | Self-care group counselling vs. Routine care | STAI: State anxiety | Postintervention  4 weeks | 35.1 | 8 | 38 | 47.1 | 10,2 | 38 | <0.001 |
|  |  | STAI: Trait anxiety |  | 37 | 8,2 | 38 | 45.6 | 9,1 | 38 | <0.001 |
| Meissner et al., 2016 | Psychotherapy with somatosensory stimulation vs. waitlist control | HADS | 3 months | -2.6 | (-4.1 to-1.2) | 31 | 0 | (-1.5 to 1.5) | 28 | 0.012 |
|  |  | STAI: Trait anxiety |  | -5.4 | (-7.6 to -3.1) | 29 | -1.3 | (-3.8 to 1.2) | 24 | 0.018 |
|  |  | HADS | 6 months | -3.2 | (-4.8 to -1.6) | 20 | -2.3 | (-4.0 to -0.7) | 19 | 0.438 |
|  |  | STAI: Trait anxiety |  | -4,9 | (-9.1 to -0.8) | 19 | -5.2 | (-9.6 to -0.8) | 17 | 0,914 |
|  |  | HADS | 24 months | -3 | (-4.4 to -1.5) | 30 | -2.5 | (-4.1 to -0.9) | 24 | 0.673 |
|  |  | STAI: Trait anxiety |  | -6.4 | (-9.7 to -3.2) | 27 | -5 | (-8.5 to -1.5) | 23 | 0.557 |
| Zhao et al., 2012 | Progressive muscle relaxation training + GnRH vs. GnRH | STAI: State anxiety | Postintervention | 33.69 | 3.74 | 42 | 42.1 | 5.37 | 45 | <0.001 |
|  |  | STAI: Trait anxiety |  | 37.76 | 3.59 | 42 | 44.98 | 6.55 | 45 | <0.001 |

Note. HADS Hospital Anxiety and Depression Scale; N: number of patients; STAI: State-trait Anxiety Inventory; VAS: Visual analogue scale; SD: standard deviation

**Supplementary Table 7.** Main Data Extracted from the Included Studies: Depression

| First author, year | Comparison | Scale | Follow-up | Treatment | | | Control | | | P- value |
| --- | --- | --- | --- | --- | --- | --- | --- | --- | --- | --- |
|  |  |  |  | Mean | SD/IC95% | N | Mean | SD/IC95% | **N** |  |
| Farshi et al., 2020 | Self-care group counselling vs. Routine care | BDI | Postintervention  4 weeks | 14.23^†^ | 11.33^†^ | 38 | 12,33^†^ | 8,89^†^ | 38 | 0.565 |
| Meissner et al., 2016 | Psychotherapy with somatosensory stimulation vs. waiting list control | HADS | 3 months | -2.5 | (-3.3 to-1.7) | 29 | 0.2 | (-0.7 to 1) | 28 | <0.001 |
|  |  | HADS | 6 months | -2.9 | (-4.3 to -1.6) | 19 | -1.9 | (-3.3 to -0.6) | 18 | 0.288 |
|  |  | HADS | 24 months | -2.9 | (-4.1 to -1.7) | 29 | -1.3 | (-2.7 to -0.0) | 24 | 0.085 |
| Zhao et al., 2012 | Progressive muscle relaxation training + GnRH vs. GnRH | HADS | Postintervention | 4.41 | 1.95 | 42 | 6.78 | 2.32 | 45 | <0.001 |
| Note. BDI: Beck Depression Inventory; HADS Hospital Anxiety and Depression Scale; N: number of patients; SD: standard deviation  ^†^ own calculation | | | | | | | | | | |

**Supplementary Table 8.** Results of Meta-analyses, Subgroups and Sensitive Analyses

| **Outcome**  **Sensitive/** **Subgroups analyses** | **K** | **N** | **Statistical Method** | **Effect Estimate** | **I^2^ (%)** | **Egger test (P- value)** | **Subgroup differences by type of intervention (P- value)** | **Subgroup differences by risk of bias (P- value)** |
| --- | --- | --- | --- | --- | --- | --- | --- | --- |
| **1. PAIN** | | | | | | | | |
| **1.1. Dyspareunia** | 3 | 160 | SMD (IV, Fixed, 95% CI) | -0.54 [-0.86, -0.22] | 0 | 0.45 | NA | 0.86 |
| By risk of bias | | | | | | | | |
| 1.1.1. Some concerns | 1 | 26 | SMD (IV, Fixed, 95% CI) | -0.61 [-1.44, 0.22] | NA |  |  |  |
| 1.1.2. High | 2 | 134 | SMD (IV, Fixed, 95% CI) | -0.53 [-0.88, -0.19] | 0 |  |  |  |
| **1.2. Dyschezia** | 2 | 88 | MD (IV, Fixed, 95% CI) | -2.90 [-4.55, -1.26] | 0 | 0.86 | NA | 0.32 |
|  |  |  | SMD (IV, Fixed, 95% CI) | -0.61 [-1.05, -0.18] | 69 |  |  |  |
| By risk of bias | | | | | | | | |
| 1.2.1. Some concerns | 1 | 34 | MD (IV, Fixed, 95% CI) | -3.50 [-5.53, -1.47] | NA |  |  |  |
| 1.2.2. High | 1 | 54 | MD (IV, Fixed, 95% CI) | \| -1.75 [-4.57, 1.07] \| \| --- \| | NA |  |  |  |
| **1.3. Pelvic pain** | 2 | 107 | MD (IV, Fixed, 95% CI) | -1.22 [-2.23, -0.22] | 0 | 0.93 | NA | 0.61 |
|  |  |  | SMD (IV, Fixed, 95% CI) | -0.42 [-0.81, -0.04] | 0 |  |  |  |
| By risk of bias | | | | | | | | |
| 1.3.1. Some concerns | 1 | 53 | MD (IV, Fixed, 95% CI) | -1.40 [-2.61, -0.19] | NA |  |  |  |
| 1.3.2. High | 1 | 54 | MD (IV, Fixed, 95% CI) | -0.84 [-2.64, 0.96] | NA |  |  |  |
| **2. QUALITY OF LIFE** | | | | | | | | |
| **2.1. Social function** | 2 | 141 | MD (IV, Random, 95% CI) | -4.47 [-26.29, 17.35] | 84 | 0.27 | NA | NA |
|  |  |  | SMD (IV, Random, 95% CI) | -0.20 [-1.09, 0.69] | 85 |  |  |  |
| **2.2. Emotional role** | 2 | 141 | MD (IV, Random, 95% CI) | -15.98 [-35.22, 3.27] | 68 | 0.07 | NA | NA |
|  |  |  | SMD (IV, Random, 95% CI) | -0.96 [-2.71, 0.79] | 96 |  |  |  |
| **2.3. Mental health** | 4 | 277 | SMD (IV, Random, 95% CI) | 1.13 [0.30, 1.96] | 90 | NA | < 0.00001 | < 0.00001 |
| Without Farshi et al. (2020) | 3 | 201 | SMD (IV, Random, 95% CI) | 0.70 [0.42, 0.99] | 0 | 0.50 |  |  |
| By intervention | | | | | | | | |
| 2.3.1. Guided intervention | 3 | 201 | SMD (IV, Random, 95% CI) | 0.70 [0.42, 0.99] | 0 |  |  |  |
| 2.3.2. Self-care counselling | 1 | 76 | SMD (IV, Random, 95% CI) | 2.56 [1.94, 3.17] | NA |  |  |  |
| By risk of bias |  |  |  |  |  |  |  |  |
| 2.3.3. Low | 1 | 76 | SMD (IV, Random, 95% CI) | 2.56 [1.94, 3.17] | NA |  |  |  |
| 2.3.4. Some concerns | 1 | 60 | SMD (IV, Random, 95% CI) | 0.57 [0.05, 1.08] | NA |  |  |  |
| 2.3.5. High | 2 | 141 | SMD (IV, Random, 95% CI) | 0.76 [0.42, 1.11] | 0 |  |  |  |
| **2.4. Physical functioning** | 2 | 141 | MD (IV, Random, 95% CI) | 8.11 [-5.61, 21.83] | 57 | 0.45 | NA | NA |
|  |  |  | SMD (IV, Random, 95% CI) | 0.39 [-0.44, 1.22] | 83 |  |  |  |
| **2.5. Physical role** | 2 | 141 | MD (IV, Random, 95% CI) | 10.98 [-7.52, 29.49] | 40 | 0.46 | NA | NA |
|  |  |  | SMD (IV, Random, 95% CI) | 0.29 [-0.38, 0.96] | 74 |  |  |  |
| **3. ANXIETY** | | | | | | | | |
| **3.1. Trait** | 3 | 216 | MD (IV, Fixed, 95% CI) | -6.63 [-8.27, -4.99] | 46 | 0.98 | 0.27 | 0.16 |
|  |  |  | SMD (IV, Fixed, 95% CI) | -1.04 [-1.33, -0.75] | 37 |  |  |  |
| By intervention |  |  |  |  |  |  |  |  |
| 3.1.1. Guided intervention | 2 | 140 | MD (IV, Fixed, 95% CI) | -6.21 [-8.01, -4.40] | 60 |  |  |  |
| 3.1.2. Self-care counselling | 1 | 76 | MD (IV, Fixed, 95% CI) | -8.60 [-12.49, -4.71] | NA |  |  |  |
| By risk of bias |  |  |  |  |  |  |  |  |
| 3.1.3. Low | 1 | 76 | MD (IV, Fixed, 95% CI) | -8.60 [-12.49, -4.71] | NA |  |  |  |
| 3.1.4. Some concerns | 1 | 53 | MD (IV, Fixed, 95% CI) | -4.10 [-7.27, -0.93] | NA |  |  |  |
| 3.1.5. High | 1 | 87 | MD (IV, Fixed, 95% CI) | -7.22 [-9.42, -5.02] | NA |  |  |  |
| **3.2. State** | 2 | 163 | MD (IV, Random, 95% CI) | -9.72 [-13.11, -6.33] | 58 | 0.12 | 0.12 | 0.12 |
|  |  |  | SMD (IV, Random, 95% CI) | -1.54 [-2.03, -1.06] | 47 |  |  |  |
| By intervention |  |  |  |  |  |  |  |  |
| 3.2.1. Guided intervention | 2 |  | MD (IV, Random, 95% CI) | -8.41 [-10.34, -6.48] | NA |  |  |  |
| 3.2.2. Self-care counselling | 1 |  | MD (IV, Random, 95% CI) | 12.00 [-16.12, -7.88] | NA |  |  |  |
| By risk of bias |  |  |  |  |  |  |  |  |
| 3.2.3. Low | 1 | 76 | MD (IV, Random, 95% CI) | -12.00 [-16.12, -7.88] | NA |  |  |  |
| 3.2.5. High | 1 | 87 | MD (IV, Random, 95% CI) | -8.41 [-10.34, -6.48] | NA |  |  |  |
| **4. Depression** | 3 | 220 | SMD (IV, Random, 95% CI) | -0.70 [-1.60, 0.20] | 90 | NA | < 0.00001 | 0.16 |
| Without Farshi et al. (2020) | 2 | 144 | MD (IV, Random, 95% CI) | -2.49 [-3.20, -1.79] | 0 | 0.94 |  |  |
|  |  |  | SMD (IV, Random, 95% CI) | -1.14 [-1.49, -0.78] | 0 |  |  |  |
| By intervention |  |  |  |  |  |  |  |  |
| 4.1. Guided intervention | 2 | 144 | MD (IV, Random, 95% CI) | -2.49 [-3.20, -1.79] | 0 |  |  |  |
| 4.2. Self-care counselling | 1 | 76 | MD (IV, Random, 95% CI) | 1.90 [-2.68, 6.48] | NA |  |  |  |
| By risk of bias |  |  |  |  |  |  |  |  |
| 4.3. Low | 1 | 76 | MD (IV, Random, 95% CI) | 1.90 [-2.68, 6.48] | NA |  |  |  |
| 4.4. Some concerns | 1 | 57 | MD (IV, Random, 95% CI) | -2.70 [-3.85, -1.55] | NA |  |  |  |
| 4.5. High | 1 | 87 | MD (IV, Random, 95% CI) | -2.37 [-3.27, -1.47] | NA |  |  |  |

Note. 95% CI: Confidence interval 95%; IV: Inverse variance; K: Studies; N: Participants; NA: Not apply; MD: Mean Difference; SMD: Standardized mean difference
